# Dynamic quality aware path planning for 6 DoF robotic arms using BiRRT and metaheuristic optimization based on B spline paths

**DOI:** 10.1038/s41598-026-37676-8

**Published:** 2026-02-22

**Authors:** Abdelrahman T. Elgohr, Maher Rashad, Eman M. El-Gendy, Waleed Shaaban, Mahmoud M. Saafan

**Affiliations:** 1 Mechatronics Engineering Department, Faculty of Engineering, Mansoura University, Mansoura, Egypt; 2 Department of Mechatronics Engineering, Faculty of Engineering, Horus University, New Damietta, 34517 Egypt; 3 Production Engineering and Mechanical Design Department, Faculty of Engineering, Tanta University, Tanta, Egypt; 4 Computers Engineering and Control Systems Department, Faculty of Engineering, Mansoura University, Mansoura, Egypt; 5 Mechanical Engineering Department, Mansoura University, El-Mansoura, 35516 Egypt; 6 Faculty of Engineering, Mansoura National University, Mansoura, Egypt

**Keywords:** Path planning, Bi-RRT, B-spline, Metaheuristic optimization, WGA, GWO, Engineering, Mathematics and computing

## Abstract

Industrial robotic arms utilized in contemporary industrial and collaborative environments must operate within increasingly congested and dynamically restricted workspaces while adhering to rigorous standards of safety, precision, and motion quality. This paper presents a two-stage framework for path planning and optimization of a 6-DOF industrial robotic arm navigating amid randomly distributed obstacles. A collision-free reference motion is initially created by integrating B-spline geometric interpolation with a bidirectional RRT-Connect planner, augmented by short-cutting and effective joint-space collision verification for a KUKA KR 4 R600 manipulator. The baseline trajectory is subsequently enhanced through two metaheuristic optimizers: a Whale Genetic hybrid algorithm (WGA) and the Grey Wolf Optimizer (GWO). These optimizers minimize a composite objective that incorporates end-effector trajectory length, joint-level energy consumption based on established motor characteristics, and trajectory smoothness measured by joint jerk. Simulation results indicate that, while the raw Bi-RRT trajectory is geometrically efficient and energy-efficient, it demonstrates excessively high jerk. The suggested enhancements based on WGA and GWO diminish the jerk index of the original Bi-RRT solution by roughly 94–96%, resulting in relatively slight increases in trajectory length and energy, while producing dynamically smooth, collision-free trajectories that adhere to all kinematic constraints. This work presents a comprehensive, implementation-ready methodology that compares sampling-based planning, and multi-objective metaheuristic optimization to produce executable, energy-efficient, and jerk-minimized motions for industrial manipulators in intricate environments.

## Introduction

Contemporary industrial manipulators function inside workcells that are increasingly dense, varied, and unpredictable. Tooling, fixtures, conveyors, human-robot co-presence, and ad-hoc inventory generate haphazard environments in which a 6-DoF arm must maneuver with precision and safety. In these contexts, motion design is a multifaceted pursuit: trajectories must be concise to maintain cycle time, energy-efficient to minimize operating costs and heat generation, and smooth at the jerk level to safeguard systems and facilitate high-gain tracking without oscillation^1,2^. Classical, single-stage motion generators serve as useful foundations; nevertheless, when confronted with irregular or unmodeled obstacle fields, they frequently result in suboptimal advances in trajectory efficiency, energy consumption, or dynamic feasibility^3^.

**Fig. 1:**
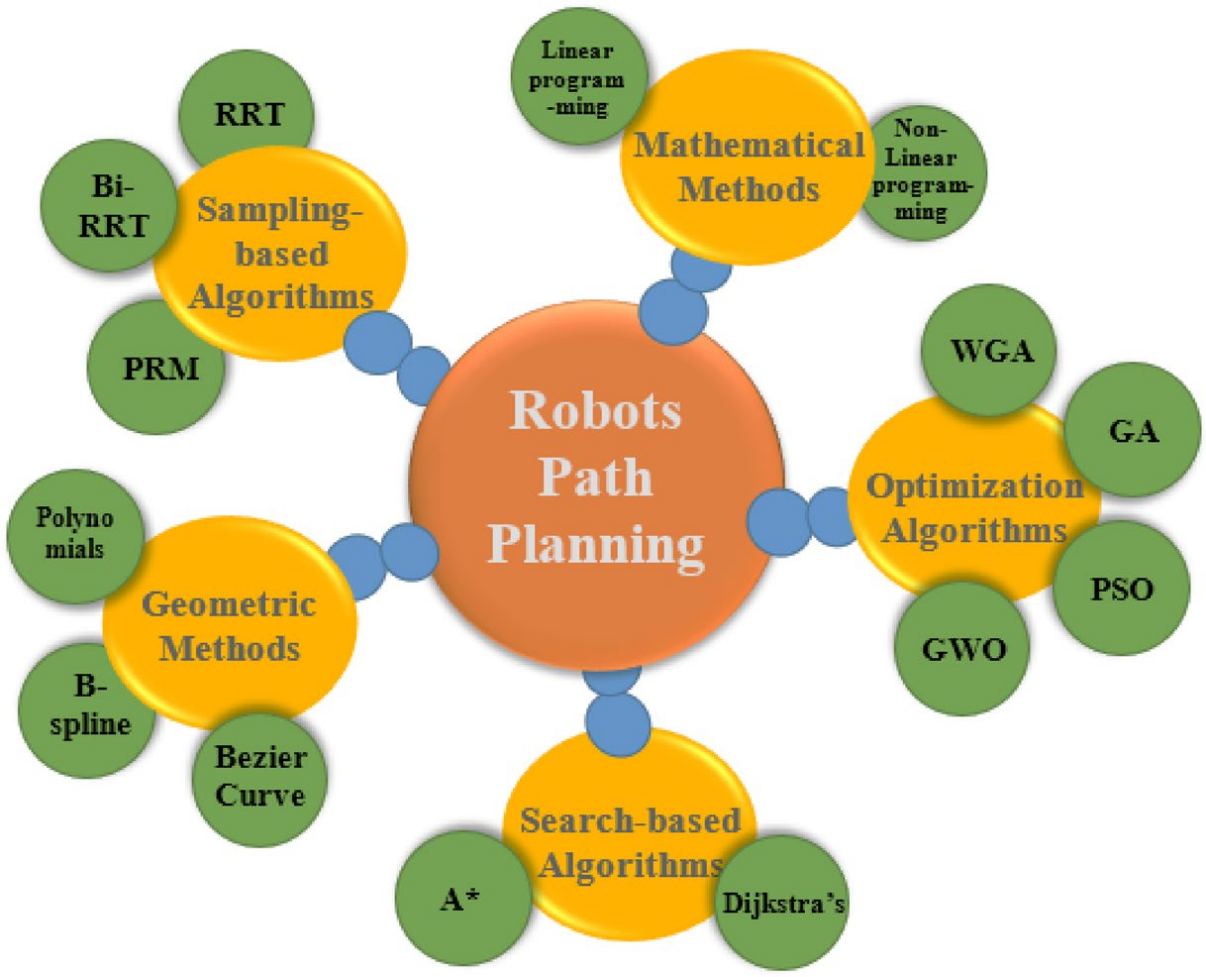
Main robot path planning techniques categories.

As categorized in Fig. 1, A longstanding history in manipulation depends on analytical trajectory families, particularly polynomial and spline parameterizations, as they provide closed-form continuity of position, velocity, and acceleration, while also being straightforward to evaluate, visualize, and constrain^4^. Splines formulations offer rest-to-rest profiles with inherent smoothness and function as reliable baselines in both manufacturing and research^5^. These curves are generally formulated in free space and subsequently modified to accommodate impediments (e.g., through potential fields or waypoint adjustments), potentially resulting in conservative timings, superfluous detours, or retrospective compromises between smoothness and trajectory length. In summary, analytical profiles are effective and streamlined; nevertheless, their efficiency in clutter is not assured in the absence of a search component^6,7^.

In contrast, sampling-based planning investigates configuration spaces without the need for explicit parameterization of complete motion, making it particularly effective for high-DoF arms in constrained environments. Variants like bidirectional trees and asymptotically optimal extensions enhance success rates and solution quality, particularly when combined with continuous collision checking and problem-aware distance measures. These planners provide viable waypoint sequences through intricate obstacle fields and can be refined through shortcutting and smoothing; nonetheless, the first outputs frequently remain inadequate concerning cycle time, energy, and jerk, necessitating a further, enhancement-focused phase^8,9^.

Directly initiating motion planning in joint space through bidirectional sampling is essential for enhancing feasibility and exploration efficiency in industrial robotic arms. In contrast to workspace-based or solely analytical trajectory generators, joint-space planning intrinsically considers the robot’s kinematic configuration, joint constraints, and coupling effects, thus minimizing the likelihood of producing infeasible or unreachable motions inside high-dimensional configuration spaces^10,11^.

Moreover, bidirectional sampling techniques, such as Bi-RRT, markedly improve convergence velocity and solution dependability by concurrently extending search trees from both the initial and target configurations. This twofold growth method enhances the likelihood of swiftly identifying legitimate connections over constricted pathways and densely populated areas, typical in industrial workcells featuring fittings, tools, and arbitrarily placed barriers. When integrated with obstacle-aware sampling biases, bidirectional joint-space planners can effectively concentrate exploration on advantageous areas of the configuration space while maintaining probability completeness^12,13^.

In contrast to unidirectional sampling, workspace-based planning, or only polynomial and spline-based methods, bidirectional joint-space sampling provides enhanced scalability and robustness in intricate situations. It offers a dependable global feasibility framework for the application of execution-aware optimization, guaranteeing that following refinement phases function on trajectories that are kinematically valid and collision-free for the entire manipulator.

This is when optimization-driven enhancement becomes critical. When initiated with an appropriate path, trajectory optimization can synchronize motion with execution-related costs, duration, energy consumption, and jerk, while adhering to constraints and clearances. Both gradient-based methods (e.g., direct collocation, shooting/DDP) and metaheuristic strategies have been employed effectively; the latter, including Genetic Algorithms^14^, Particle Swarm Optimization^15^, Differential Evolution, Simulated Annealing, Ant Colony Optimization, Artificial Bee Algorithms, and Grey Wolf/Whale family hybrids, provide derivative-free global exploration and resilience to nonconvex penalties prevalent in collision-aware planning^14,16,17^. Empirical evidence indicates that metaheuristics effectively shorten pathways and diminish jerk compared to unoptimized polynomial baselines, while enhancing efficiency over geometric plans in clutter, albeit with additional computational demands.

## Related works

Recent surveys and book chapters offer a comprehensive perspective on path planning and trajectory planning for industrial robotics, highlighting that effective implementation necessitates a compromise among feasibility, efficiency, smoothness, and application-specific limitations. Boscariol et al. present a practical examination of “special robotic operations” and demonstrate that industrial tasks, such as welding, spray painting, AGV-enabled logistics, and constrained workcells, impose complex requirements on planning pipelines that extend beyond mere shortest-trajectory feasibility^18^. Ugwoke et al. recently conducted a simulation-based review of classical, heuristic, and metaheuristic path planning algorithms, emphasizing the increasing utilization of population-based metaheuristics for nonconvex planning challenges and the enduring trade-offs between solution quality and computational time^19^.

Inspired by these viewpoints, our research employs a seed-and-refine framework specifically designed for industrial manipulators operating in cluttered workcells, addressing a gap that remains insufficiently explored in numerous manipulator-centric studies: the explicit optimization of actuation-related execution costs (joint-energy and jerk) as primary objectives, rather than considering smoothness as a mere secondary outcome of geometric post-processing.

Recent research in sampling-based motion planning emphasizes that enhanced sampling and post-processing can significantly enhance feasibility and geometric quality in cluttered environments. Direction-adaptive bidirectional growth and central multi-node expansion enhance connectivity in high-dimensional spaces and diminish the variance of raw trees, while pruning suboptimal branches through informed (ellipsoidal) sampling further focuses computation in areas where solution quality is most likely to improve^20–23^. Methods that incorporate singularity awareness and spline regularization at the planning level demonstrate that “implement ability” can be advanced upstream—meaning planners can generate pathways that are more aligned with controller-ready trajectories instead of relegating all smoothing to a subsequent step^24^. These investigations reveal a consistent trend: a preference for guidance (heuristics or geometry) that minimizes superfluous exploration while maintaining completeness in practical scenarios.

Hybrid, field-guided methodologies serve a supplementary function by incorporating analytical structure into the search process. Whole-arm potential-field formulations translate workspace obstacles into configuration space, facilitating the rapid exclusion of dangerous areas; when integrated with graph search or sampling methods, they assist in circumventing local minima and diminishing oscillations that typically afflict Artificial Potential Field (APF)-only approaches^25–28^. Significantly, goal-biased RRT–APF and APF-assisted secondary planning at collision points demonstrate consistent improvements in planning time and clearance in dense environments; however, the optimization objectives predominantly focus on geometric factors (distance, clearance), with smoothness achieved indirectly through polynomials or splines rather than as an explicit dynamic objective.

A second, more significant approach is “seed-and-refined”: using a rapid global planner, followed by the application of nonconvex optimization to adjust time and curvature. Deterministic homotopy continuation utilizes workspace topology to provide consistent solutions within constrained memory and time limits, whereas particle-swarm timing and quintic parameterization demonstrate that straightforward, well-optimized adjustments can significantly decrease execution time post-sampling^20,29^. Metaheuristics implemented at the trajectory level and exemplified on industrial robotic arms—such as Informed-RRT* integrated with local trajectory optimization and quintic timing, or direct whale-based search, furnish further evidence that global-local combinations can surpass purely geometric baselines in terms of length and smoothness within constrained three-dimensional environments^30,31^. The overarching message is that the usefulness of optimization is greatest when it functions on a viable seed and directly addresses execution-relevant criteria.

Ultimately, deployment-focused contributions prioritize resilience to layout, fixtures, and singularity neighborhoods. Digital planners for welding lines minimize human involvement through grid abstractions and posture modifications; multi-node expansion and reconstructed informed sampling decrease sensitivity to initial conditions; and singularity-aware planners, regularized with B-splines or progressive inverse kinematics, stabilize motion in proximity to challenging configurations in real robots^21,24,32,33^. These efforts implicitly demonstrate a transition from “shortest-trajectory” reasoning to “plant-aware” strategizing. Nevertheless, a persistent disparity exists: execution costs associated with actuators—particularly joint-energy and jerk, are seldom incorporated as primary objectives. This disparity prompts our bifurcated approach: Bi-RRT seeding ensures dependable feasibility in randomly cluttered, user-defined environments, succeeded by metaheuristic refinement (WGA/GWO) that explicitly optimizes a composite objective encompassing length, energy, and jerk, thereby converting geometric feasibility into trajectories that are significantly more execution-ready.

Table 1 compares recent manipulator planning studies by seeding strategy, refinement, objectives, and deployment concerns; the table highlights a persistent gap in execution-oriented costs (energy, jerk) and motivates our two-stage approach (Bi-RRT seeding + metaheuristic refinement with energy- and jerk-aware objectives) in randomly cluttered, user-specified environments.

**Table 1:** Literature comparison for collision-free manipulator planning across seeding, refinement, objectives, and deployment factors.

Refs.	Seed class	Refinement stage	Objective targets	Environment focus	Validation	Reported gains/notes
^20^	Sampling (bi-RRT*)	PSO timing	Trajectory length, T (run time)	Static clutter (industrial frames)	Sim	−38.7% C-space length, − 57.4% gen time; −45.2% run time
^22^	Sampling (RRT variants)	Implicit (iterative pruning)	T (plan time), trajectory length	Randomized (2D) + UR5 sim	VREP sim (UR5)	−28.9% compute vs. Informed-RRT*; faster than Bi-RRT*
^21^	Sampling (RRT*)	Implicit (multi-tree)	Time, trajectory length	High-dof randomized	Sim	−69.8% calc time; +6.1% trajectory quality
^23^	Structured marching	Post-smoothing	Time	Static & dynamic	Real robot (6-dof)	0.51–1.63 s (static); 0.62–0.88 s (replan)
^24^	Structured marching	B-spline	Trajectory length, smoothness (spline)	Cartesian scenes	Sim + real scenarios	Shorter, smoother trajectories; avoids singularities
^32^	Roadmap (lazy-PRM)	Local posture tweaks	Time, feasibility	Online welding (open cell)	Sim + real app	Practical online collision avoidance; reduced human effort
^25^	Analytical (APF)	–	Trajectory length (geometric), feasibility	Multi-station welding	Real (MA1440)	Intuitive avoidance; classic APF limitations persist
^28^	Hybrid (APF + RRT)	Secondary plan at collisions	Time, feasibility	With/without obstacles	Sim	−32.12%/−33.43% planning time
^26^	Hybrid (A*+APF)	Posture adjustment	Trajectory length, feasibility	Varied scenes	AUBO-i10 experiments	Smooth, obstacle-free motion on hardware
^27^	Hybrid (RRT + APF)	Sub-targets then config-space planning	Trajectory length, feasibility	Constrained grasping	DOBOT CR5 (MATLAB)	Escapes APF local minima; task-specific demo
^29^	Deterministic global	Global solution trajectory	Trajectory length, feasibility	Narrow corridors + general	Real (CRS catalyst-5)	Ms (classical arms), s/KB (hyper-redundant)
^31^	Sampling + local opt.	Refine + timing	Time, trajectory length, smoothness (poly)	Highly dynamic, constrained	6-dof sim	Faster convergence; whole-arm avoidance
^33^	Segmentation + IK	Recursive migration	Time, trajectory length, stability	Multi-obstacle; real exec	Real (6-dof)	0.017 s for 2D trajectory; robust near singularities
^30^	Metaheuristic (WOA)	WOA	Trajectory length	With/without obstacles	Case study (KR4 R600)	> 30% reduction in EE travel distance

### Statement of the problem

Notwithstanding these advancements, a deficiency persists for a cohesive, execution-focused two-stage framework that (i) initiates motion in joint space utilizing bidirectional sampling tailored to user-defined random impediments; (ii) optimizes a singular cost that collectively assesses end-effector compactness, actuator energy utilizing established constant motor powers, and jerk while imposing explicit penalties for collisions and limits—advancing beyond the primarily geometric objectives found in APF/A* hybrids and numerous refinement stages; and (iii) is validated on industrial-grade manipulators equipped with engineer-ready tools (MATLAB, explicit obstacle input/output, 3-D visualization) and statistically robust protocols. This study fulfills this requirement by integrating a joint-metric RRT with shortcutting for swift feasibility, alongside metaheuristic trajectory refinement (WGA and GWO) that directly optimizes the execution-relevant objective.

This research presents a two-stage, execution-aware trajectory planning approach for 6-DoF industrial manipulators functioning in crowded settings to tackle these problems. Initially, joint-space, spline-guided bidirectional Rapidly-Exploring Random Tree (Bi-RRT) with shortcutting is utilized to effectively navigate high-dimensional configuration spaces and provide collision-free feasibility for the entire manipulator, rather than solely the end-effector. This sampling-based phase is specifically engineered to manage intricate obstacle configurations and confined pathways frequently encountered in industrial workcells.

In the second phase, the viable trajectory derived from the global planner is enhanced by metaheuristic optimization methods, specifically the Whale Genetic Algorithm (WGA) and the Grey Wolf Optimizer (GWO). This refinement phase specifically tackles the multi-objective aspect of industrial trajectory planning by maximizing a composite cost function that includes trajectory length, joint-level energy consumption, and joint-space jerk. The proposed methodology directly targets execution-relevant dynamic measures to build trajectories that are collision-free, smoother, more energy-efficient, and optimized for precise tracking and long-term mechanical durability.

In contrast to current methods that focus mainly on geometric feasibility or shortest-trajectory criteria, the proposed approach offers a systematic solution that integrates global feasibility with execution quality, rendering it especially appropriates for real industrial robotic arms constrained by stringent kinematic and actuation limitations.

### Main contributions

- Two-stage hybrid for random clutter. A RRT + shortcutting global stage in joint space with a joint-range-weighted metric and continuous collision checks, followed by trajectory optimization with timing, designed to bridge feasibility and execution quality.
- Summative, dynamics-aware cost. A single objective blending trajectory length, constant-power energy, and jerk, plus penalties for collisions and joint/velocity/acceleration limits—aligning optimization with metrics that matter on hardware.
- Dual-metaheuristic comparison under identical constraints. A fair study of WGA and GWO on the same decision variables, bounds, and penalties to expose exploration–exploitation trade-offs and convergence behaviors.
- Reproducible MATLAB pipeline with explicit obstacle inputs. A complete implementation for user-defined obstacle positions and dimensions, 3D visualizations, and standardized outputs for statistical analysis.

### The paper is structured as follows

Section 2 delineates the proposed methodology and materials, commencing with the modeling of the 6-DoF robotic arm and an obstacle-laden workspace, followed by the B-spline-based time-parameterized trajectory generation and the Bi-RRT with short-cutting as the global, collision-free path construction phase. Subsequently, it formulates a multi-objective optimization framework that integrates trajectory length, joint energy consumption, and jerk, with specific subsections elucidating the implementation of the Whale Genetic algorithm (WGA) and Grey Wolf Optimizer (GWO). Section 3 presents and examines the quantitative findings. the robotic model and its constraints, the selected performance metrics, the baseline B-spline and Bi-RRT results, the optimized trajectories derived from WGA and GWO, and an extensive comparative analysis emphasizing the trade-offs between feasibility, energy efficiency, and smoothness. Section 4 closes the study by summarizing the principal findings and suggesting future research avenues for human–robot collaboration scenarios augmented by vision and EEG-based intent sensing.

## Materials and methods

**Fig. 2:**
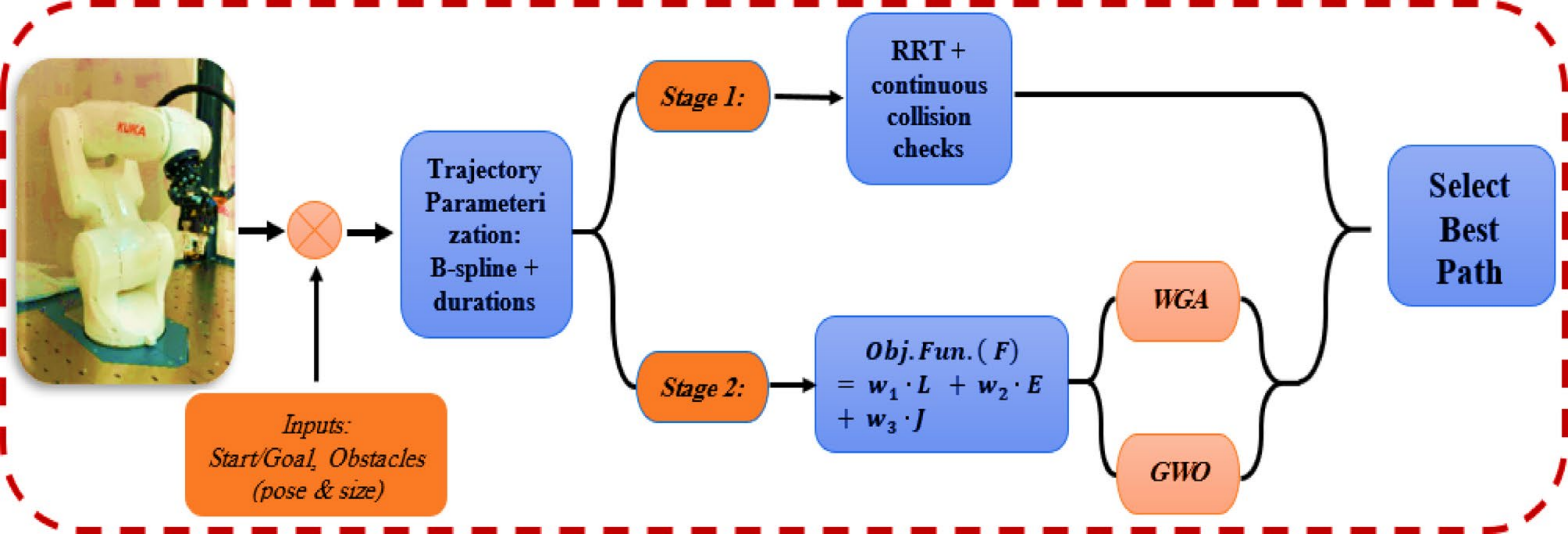
Proposed study methodology pipeline.

The pipeline of the study methodology, which shown in Fig. 2, initiates with explicit scene inputs. the robot model (6-DoF with joint, velocity, and acceleration constraints), initial and target configurations, and user-defined obstacles given by their poses and dimensions. The barriers are transformed into collision geometries with a safety margin and are examined through continual segment checks during the design process. A succinct, implementable framework of the motion is subsequently generated utilizing a bidirectional RRT with a joint-range-weighted distance metric. This decision enhances exploration in the most “efficient” joint directions and facilitates advancement via constricted pathways. The sampler initiates two trees from the start and goal, interlinks them with collision-free segments, and produces a sparse sequence of joint-space waypoints that adhere to link-obstacle separation requirements. A time-parameterized trajectory template is concurrently developed by B-spline segments between waypoints and designating beginning segment durations; this results in closed-form derivatives up to jerk and a uniform time grid for subsequent evaluation and visualization.

The seeded trajectory is optimized by reducing a singular execution-focused objective. Where represents the end-effector trajectory length, quantifies actuator energy utilizing established constant motor powers (prioritizing reduced active time while maintaining feasibility), and denotes the integral of joint-space jerk to enhance controller-friendly smoothness. Penalties and clamping are applied during evaluation to enforce collision proximity and constraints on joint, velocity, and acceleration. Two metaheuristics, WGA and GWO, function on identical decision variables (control points and segment durations), each iteratively generating candidate trajectories that are evaluated by through continual collision assessments. The optimal candidate from both optimizers is subsequently chosen as the final trajectory and presented alongside 3-D scene visualizations and kinematic profiles; this dual-stage design integrates rapid feasibility from the sampler with a global, derivative-free search to produce shorter, smoother, and more energy-efficient motions in randomly cluttered environments.

### B-spline based path planning

B-splines are a category of piecewise polynomial curves characterized by compact support and adjustable smoothness, which have emerged as a common instrument for robotic trajectory development owing to its geometric and numerical benefits^34^. A B-spline curve is characterized by a degree, a nondecreasing knot vector, and a collection of control (or interpolation) points. B-splines, in contrast to global polynomials, exhibit local control; altering a single control point affects the curve solely within a restricted parameter range^35^. Furthermore, they maintain uniform $\:{C}^{p-1}$ continuity at internal knots when the knot multiplicity is one. These attributes enable the planner to construct intricate trajectories that maintain smoothness, impose boundary conditions without compromising internal regularity, and adhere to geometric constraints such as convex-hull and variation-diminishing properties, thereby stabilizing inverse-kinematics (IK) tracking and minimizing oscillations. Subsequently, it employed a cubic (=3)$\:{C}^{2}$ B-spline interpolant utilizing a limited, obstacle-aware collection of waypoints; this formulation can be extended to higher degrees if enhanced smoothness is necessary^36,37^.

Given a start end-effector (EE) position $\:{X}_{s}=({X}_{i},\:{Y}_{i},\:{Z}_{i})\:$and a goal position $\:{X}_{g}=({X}_{f},\:{Y}_{f},\:{Z}_{f})$, as well as three spherical obstacles $\:{\left\{\right({C}_{k},\:{d}_{k}\left)\right\}}_{k=1}^{3}$with centers $\:{C}_{k}\in\:\:{\mathbb{R}}^{3}$ and diameters $\:{d}_{k}$ (radii $\:{r}_{k}={d}_{k}/2$), it first had constructed a small set of detour waypoints that guarantee geometric clearance with a safety margin $\:\delta\:>0$. Let $\:d={X}_{g}-{X}_{s}$​ and consider the straight segment $\:X\left(t\right)={X}_{s}+t\cdot\:d,\:t\in\:\left[\mathrm{0,1}\right]$. For each obstacle it had computed the clamped projection of its center onto the segment and the corresponding closest point^38^,

1 $$\:{t}_{k}^{\mathrm{*}}\text{}=cli{p}_{\left[\mathrm{0,1}\right]}\text{}\left(\frac{{\left({C}_{k}\text{}-{X}_{s}\text{}\right)}^{\mathrm{\:}}\cdot\:d}{{\parallel{d}\parallel}^{2}}\text{}\right),\:\:{P}_{k}^{\mathrm{*}}\text{}={X}_{s}\text{}+{t}_{k}^{\mathrm{*}}\text{}\cdot\:d$$

If the distance $\:{d}_{k}^{*}=\parallel{P}_{k}^{*}-{C}_{k}\parallel$ is smaller than the inflated radius $\:{r}_{k}+\delta\:$, it placed a single detour waypoint radially away from the obstacle^39^,

2 $$\:{w}_{k}\text{}={P}_{k}^{\mathrm{*}}\text{}+\left[\left({r}_{k}+\delta\:\right)-{d}_{k}^{\mathrm{*}}\text{}+\epsilon\:\right]\frac{{P}_{k}^{\mathrm{*}}-{C}_{k}}{\parallel\:{P}_{k}^{\mathrm{*}}-{C}_{k}\text{}\parallel\:\text{}}$$

where $\:\epsilon\:$ is a small slack (e.g., 0.02 m) that compensates for spline curvature between waypoints. Among all candidates, at most five with the deepest penetrations are retained and ordered by $\:{t}_{k}^{*}$​, forming $\:W=\{{X}_{s},\:{w}_{1},\:.\:.\:.\:,{w}_{m},{X}_{g}\}$ with $\:m\le\:3$.

Then, it fitted a cubic $\:{C}^{2}$ B-spline through $\:W$. Denote the interpolated points (used as control points in our implementation) by $\:{\left\{{P}_{i}\right\}}_{i=0}^{n}$​, the degree by =3, and the nondecreasing knot vector by$\:\:U$. The geometric trajectory is.

3 $$\:C\left(u\right)=\sum\:_{i=0}^{n}{N}_{i,p}\left(u\right)\cdot\:{P}_{i}\text{},\:\:u\in\:[{u}_{0}\text{},{u}_{n}\text{}]$$

where $\:{N}_{i,p}\left(u\right)$ are the Cox–de Boor basis functions. Directly time-parameterizing by $\:u$ can create speed bias because $\:u$ is not proportional to arclength. To obtain a physically meaningful and numerically stable time parameter, we reparametrize by arclength. The cumulative arclength is.

4 $$\:S\left(u\right)=\underset{{u}_{0}}{\overset{u}{\int\:}}\parallel\text{}{C}^{{\prime\:}}\left(\xi\:\right)\parallel\:d\xi\:,\:\:\:\:\:{S}_{tot}=S\left({u}_{n}\right)$$

For a fixed motion budget $\:{T}_{tot}=2\:sec$, it has enforced constant-speed traversal via the linear length–time map, and the monotone inverse computed numerically over a dense geometric pre-sampling of $\:C$. The time-parameterized End-Effector ($\:EE$) trajectory is then.

5 $$\:X\left(t\right)=C\left(u\left(t\right)\right),\:\:\:\:\:\:\:\:\:0\le\:t\le\:{T}_{tot}$$

Tracking of $\:X\left(t\right)$ in joint space is performed with inverse kinematics and a fixed desired tool orientation $\:{R}_{d}$​ (or a path-tangent orientation field when required). At each sample $\:{t}_{k}$​ the pose error is given by.

6 $$\:e={\left[{{e}_{p}}^{T}\:{{e}_{o}}^{T}\right]}^{T}\:\in\:\:{\mathbb{R}}^{6}$$

where $$\:{e}_{p\:}=x\left({t}_{k}\right)-x\left(q\right)\:and\:{e}_{o}=\omega\:\hspace{0.17em}\theta\:$$.

A damped least-squares update with joint-limit clamping is used,

7 $$\:\varDelta\:q={J}^{\mathrm{\:}}{\left(J{J}^{\mathrm{\:}}+{\lambda\:}^{2}{I}_{6}\text{}\right)}^{-1}e,\:\:\:\:\:\:\:\:q\leftarrow\:{\varPi\:}_{\left[{q}_{min}\text{},{q}_{max}\text{}\right]\text{}}(q+\varDelta\:q)$$

where $\:J$ is the geometric Jacobian, $\:\lambda\:>0$ the damping factor, and Π the elementwise projection onto joint limits. Warm-starting each solve from the previous $\:q$ ensures continuity and accelerates convergence.

Although numerical outcomes are deferred to the Results and Discussion, we define the scalar functionals extracted from the time-parameterized trajectory, as they will be central to later evaluation and optimization. The geometric trajectory length is.

8 $$\:L={\int\:}_{0}^{{T}_{tot}}\parallel\dot{X}\left(t\right)\parallel\:dt$$

The energy proxy based on known per-radian constants $\:{e}_{i}$​ for the six actuators is.

9 $$\:E=\sum\:_{i=1}^{6}{e}_{i}{\int\:}_{0}^{{T}_{tot}}\left|\dot{{q}_{i}}\left(t\right)\right|\:dt$$

and the smoothness term is the joint-space jerk functional.

10 $$\:J={\int\:}_{0}^{{T}_{tot}}{\parallel\stackrel{\dots}{q}\left(t\right)\parallel}^{2}\:dt$$

### RRT-based global path planning (Bi-RRT with short-cutting)

**Fig. 3:**
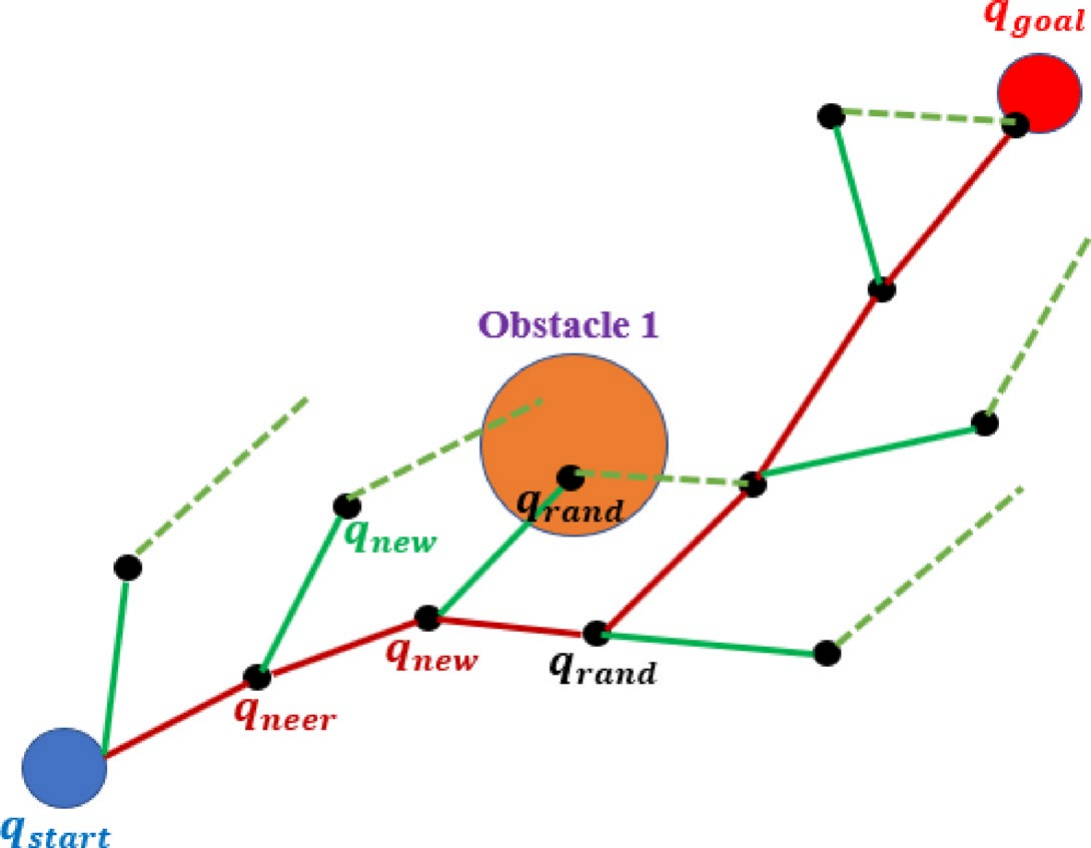
Bi-RRT goal solution research technique.

Rapidly-Exploring Random Trees (RRT) are a category of sampling-based motion planners that expand trees directly within the robot’s configuration space by periodically selecting random targets and directing the nearest node towards them while conducting local collision assessments. The straightforward “sample-and-extend” process engenders a space-filling exploration bias favoring extensive unexplored areas, enabling RRT to scale effectively in high-dimensional, nonconvex spaces characteristic of articulated manipulators without the necessity of building an explicit free-space representation, as simulated in Fig. 3^40^. The probabilistic completeness of RRT guarantees that, with sufficient samples, a viable path will be identified if it exists inside the defined domain, while its anytime characteristic yields useable, if unsatisfactory, solutions promptly, which can be refined through post-processing. Due to the lazy validation of edges through rapid local checks, RRT inherently supports intricate geometries, task-space constraints, joint limitations, and specialized distance metrics; it also exhibits strong parallelization capabilities and allows for efficient variations as Bi-RRT for expedited connections and RRT* for asymptotic optimality. The characteristics of RRT render it particularly appealing as a global feasibility engine in manipulator planning. it can swiftly identify collision-free homotopy classes amidst obstacles, generating a joint-space trajectory that subsequent smoothers and optimizers can enhance^21,41^.

This phase ensures global feasibility in complex environments by creating a collision-free joint-space trajectory that adheres to the nominal B-spline path outlined in subsection 2.1, while ensuring obstacle avoidance for the entire manipulator, not solely the end-effector. A bi-directional rapidly-exploring random tree (Bi-RRT) is utilized due to its scalability in high-dimensional configuration spaces and its probabilistic completeness; if a solution exists inside the constrained domain, the likelihood of discovering one approaches certainty as the number of samples increases. The current planner is spline-guided, utilizing a nominal B-spline to direct samples toward a tubular corridor surrounding the intended route, so expediting the connection between the start and objective while maintaining the capacity to navigate around obstacles.

For configuration space, metric, and spline-biased sampling, Planning proceeds in the 6-DOF configuration space $\:C=\left[{q}_{min},\:{q}_{max}\right]\in\:{\mathbb{R}}^{6}$. To reflect heterogeneous joint ranges, nearest-neighbor queries use a range-weighted metric. Let $\:{{r}_{i}=q}_{max,\:i}-{q}_{min,\:i}$ ​ and $\:W=diag({{r}_{1}}^{-1},\:\dots\:,\:{{r}_{6}}^{-1})$. The distance between $\:{q}_{start},\:{q}_{goal}\in\:C$ is.

11 $$\:d({q}_{start}\text{},{q}_{goal}\text{})={\parallel\text{}W\left({q}_{start}\text{}-{q}_{goal}\text{}\right)\text{}\parallel}_{2}\text{}$$

Samples are obtained through a mixture policy. with probability _*tube*_, a point on the B-spline is randomly selected based on arc-length, inverse kinematics (IK) yields a proximate configuration (with multiple attempts if necessary), and zero-mean noise (restricted to) is introduced; with probability 1−_*tube*_, a uniform sample within is drawn (incorporating a slight goal bias ). This spline-tube bias maintains global research while focusing efforts on areas where a solution is probable^42^.

Obstacles are represented as user-defined spheres {(,)}; each sphere is enlarged by a safety margin to account for link thickness and clearance. Edges undergo validation through a broad-to-narrow examination. (i) Joint-space linear interpolation is discretized at a resolution of Δ; (ii) Forward kinematics position link meshes along each sub-segment; (iii) A rapid collision checker (FCL) evaluates against the inflated spheres. An edge is deemed acceptable only if all sub-segments are devoid of collisions and adhere to joint constraints^43^.

For bidirectional expansion and connection, two trees $\:{T}_{start}\:and\:{T}_{goal}$ are initialized at $\:{q}_{start}\:and\:{q}_{goal}$​, obtained via IK at the spline’s start and goal poses. At each iteration, a random target $\:{q}_{rand}$ is drawn by the mixture sampler. In the Extend step, the nearest vertex $\:{q}_{near}$​ (under Eq. 11) is advanced toward $\:{q}_{rand}$​ by a step-size-limited steer.

12 $$\:{q}_{new}\mathbf{}={q}_{near}\mathbf{}+\gamma\:\left({q}_{rand}\mathbf{}-{q}_{near}\mathbf{}\right),\:\:\gamma\:=\mathrm{min}\left(1,\:\frac{\eta\:}{d\left({q}_{start}\text{},{q}_{goal}\text{}\right)}\right)$$

with user step $\:\eta\:>0$ in the weighted metric. If the edge ($\:{q}_{near}\approx\:{q}_{new}$) is valid, $\:{q}_{new}$​ is inserted. Immediately thereafter, a connect attempt grows the *other* tree toward $\:{q}_{new}$​ by repeated application of Eq. 12 until collision intervenes or the node is reached. If the two trees meet within a tolerance $\:\epsilon\:$ (under Eq. 11), their parent chains are concatenated to yield a raw joint-space path.

The raw path often contains redundant waypoints. A short-cutting pass selects non-adjacent pairs $\:({q}_{i},{q}_{j}),\:\:j>i+1$, and replaces $\:\{{q}_{i+1},\dots\:,{q}_{j-1}\}$ by the straight interpolation if it is collision-free under the same validation. Iteration continues for a fixed budget or until no further improvements arise. The final joint sequence is then mapped back to the task space to verify adherence to the splines time law (constant end-effector speed over the fixed horizon. In the proposed pipeline, the optimizer refines only *geometric* detour positions within pre-computed safety corridors, thereby preserving feasibility while improving length, energy, and jerk.

Bi-RRT terminates upon connection, iteration/time budget, or failure to improve. With uniform components in the sampler and fixed *η*, the method is probabilistically complete. Practical settings used in this work are. $\:\eta\:\in\:[0.05,\:0.2]$ (under Eq. 11), goal bias $\:{p}_{g}\in\:[0.05,\:0.2]$, spline-tube mixture $\:{p}_{tube}\in\:[0.5,\:0.8]$, and a connect resolution $\:\varDelta\:s$ matched to the smallest link length. Nearest-neighbor search is accelerated in whitened coordinates $\:\stackrel{\sim}{q}=Wq$, reducing Eq. 12 to a standard Euclidean query. This guided Bi-RRT thereby delivers a topologically valid, collision-free joint-space route that honors the B-spline intent.

To implement the global planner, a bidirectional RRT-Connect is developed in the joint space utilizing a weighted metric corresponding to the ranges of the joints, with edge feasibility confirmed through segment-wise collision assessments (capsule-sphere test via forward kinematics). Upon the connection of the trees, the unrefined joint trajectory is streamlined by collision-preserving shortcuts, aligned with end-effector waypoints, and temporally parameterized using equal-arclength Catmull/B-spline across a predetermined 2 s horizon. The resultant reference is subsequently monitored by a damped least square, position-only inverse kinematics, constrained by joint restrictions. The pseudocode, in Algorithm 1, encapsulates the pipeline.

**Algorithm 1:**
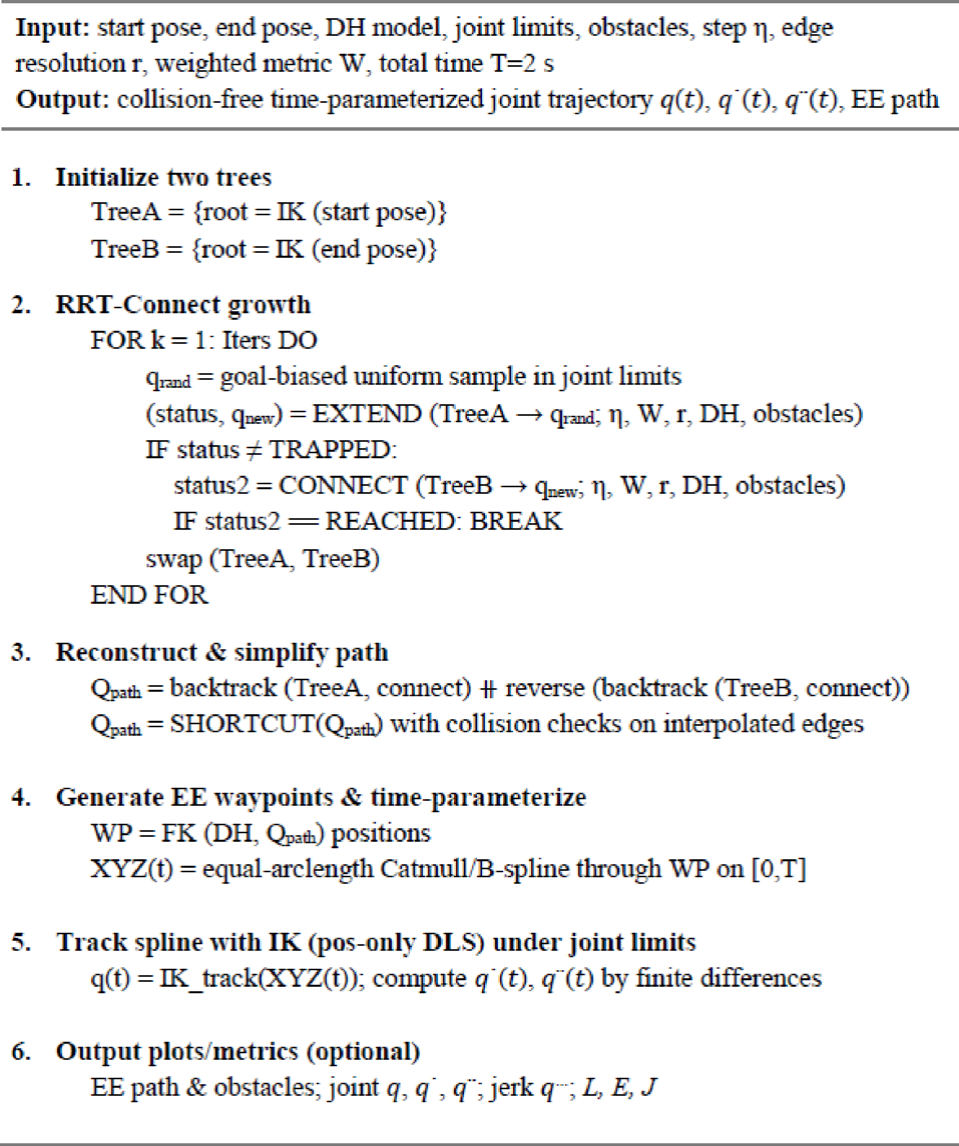
Bi-RRT (Connect) + Short-Cutting + B-spline tracking.

### Optimized path planning

Optimization is employed to transform a just viable motion into one that is systematically enhanced concerning several, potentially conflicting, performance metrics. This study employs optimization subsequent to the construction of a smooth, collision-aware B-spline trajectory, enabling the search to occur inside a constrained, feasibility-preserving design space instead of the entire, nonconvex domain of collision-free motions. Decoupling feasibility, managed geometrically through splines and diversions, from performance refinement, addressed algorithmically, enhances convergence stability and diminishes the computational load of repetitive collision checks^44^.

### Collision-free corridor construction

To ensure that trajectory optimization preserves collision-free feasibility, the proposed framework restricts all geometric modifications to predefined collision-free corridors constructed around the initial Bi-RRT–seeded trajectory. These corridors are generated based on the local geometry of the obstacles and a user-defined safety margin, and serve as bounded regions within which intermediate waypoints may be adjusted during optimization.

Let the workspace obstacles be represented as a set of inflated spheres _k_, each defined by a center *c*_*k*_ ∈ ℝ³ and an effective radius *R*_*k*_ *= r*_*k*_ *+ δ*, where *r*_*k*_ is the physical obstacle radius and *δ* is a safety margin accounting for link thickness and clearance. Consider an initial collision-free end-effector trajectory parameterized by *x(t)*, obtained from the spline-guided Bi-RRT stage. For each intermediate detour waypoint *w*_*i*_ along this trajectory, the minimum distance to the obstacle set is computed as:

13 $$\:{d}_{i}\:=\:mi{n}_{k}\:(\Vert\:{w}_{i}-\:{c}_{k}\Vert\:\:-\:{R}_{k})$$

If *d*_*i*_ *> 0*, the waypoint lies within the free space, and a local collision-free corridor can be defined as a spherical region centered at *w*_*i*_ with radius *ρ*_*i*_, where: *ρ*_*i*_ *= α · d*_*i*_,* 0 < α ≤ 1*, and α is a scaling factor (typically α ∈ [0.5, 0.8]) that provides a conservative bound to account for spline curvature between adjacent waypoints.

During optimization, each waypoint adjustment $\:\varDelta\:{w}_{i}$ is constrained such that $\:\Vert\:\varDelta\:{w}_{i}\Vert\:\:\le\:\:{\rho\:}_{i}$, ensuring that the modified waypoint remains within the collision-free corridor. After each update, a projection step is applied: if a waypoint violates the inflated obstacle boundary, it is radially projected outward along the local obstacle normal until the clearance constraint is restored.

Following waypoint modification, a cubic C² B-spline is re-interpolated through the updated waypoint set, and collision checking is performed along the resulting continuous trajectory using discretized sampling and whole-arm collision verification. This corridor-based restriction significantly reduces the dimensionality of the feasible search space, accelerates convergence, and guarantees that the optimization process operates exclusively within collision-free regions.

The end-effector (EE) curve generated in by B-spline is re-parameterized according to arc length, and a constant-speed temporal law is implemented by design. Let () represent the cubic ^2^ B-spline with parameter ∈ [_0_, _1_]. The cumulative arc length is defined as follows.

14 $$\:S\left(u\right)={\int\:}_{{u}_{0}}^{{u}_{1}}\parallel{C}^{{\prime\:}}\left(\xi\:\right)\parallel\:d\xi\:,\:\:\:\:\:\:\:\:{S}_{tot}\text{}=S\left({u}_{1}\text{}\right)$$

A linear length–time map $$\:S\left(t\right)=\left(\raisebox{1ex}{${S}_{tot}$}\!\left/\:\!\raisebox{-1ex}{$T$}\right.\right)\cdot\:t$$ on $$\:t\in\:[0,\:T]$$ is imposed and the spline parameter is recovered by the monotone inverse $$\:u\left(t\right)={S}^{-1}\left(s\right(t\left)\right)$$, yielding the time trajectory

15 $$\:x\left(t\right)=C\left(u\left(t\right)\right),\parallel\dot{x}\left(t\right)\parallel=\left(\raisebox{1ex}{${S}_{tot}$}\!\left/\:\!\raisebox{-1ex}{$T$}\right.\right)\:\:for\:all\:t\in\:[0,\:T]\:$$

This ensures a consistent Cartesian speed, unaffected by later optimization; no time-scaling variable is introduced, and speed constancy is independent of penalties.

Performance is evaluated based on three scalar functionals derived from the time-parameterized motion and the associated joint trajectory (), which is produced by inverse kinematics with a predetermined tool orientation^45^. The geometric length (*L*) corresponds to the entire arc length, the energy proxy (*E*) represents energy consumption as a per-radian cost aggregated across joints, and smoothness is measured by the joint-space jerk functional (*J*). A normalized, weighted goal is minimized to equilibrate diverse scales while maintaining interpretability of the possible seed.

16 $$\:Obj.\:Func.\:={{w}_{L}\cdot\:\left(\frac{L}{{L}_{0}}\right)+w}_{E}\cdot\:\left(\frac{E}{{E}_{0}}\right)+{w}_{J}\cdot\:\left(\frac{J}{{J}_{0}}\right)$$

where, $$\:{w}_{L}\boldsymbol{},{w}_{E}\boldsymbol{},{w}_{J}\boldsymbol{}\ge\:0,\:\:{w}_{L}\boldsymbol{}+{w}_{E}\boldsymbol{}+{w}_{J}\boldsymbol{}=1$$

Here, _0_, _0_, and _0_ represent baselines recorded during the initial spline-tracked motion. The modifications are limited to convex, collision-free corridors defined by local obstacle geometry, ensuring that feasibility is inherently maintained and re-interpolation remains appropriately structured.

Strict constraints are enforced on boundary conditions, joint limitations, and minimum clearance, and are evaluated on a consistent time grid. Let () represent the signed clearance to the union of inflated spherical obstacles of radius +. The limitations state as.

17 $$\:x\left(0\right)={X}_{s}\text{},\:\:\:\:\:\:x\left(T\right)={X}_{g}\text{},\:\:\:\:\:{q}_{min}\text{}\le\:q\left(t\right)\le\:{q}_{max}\text{},\:\:\:\:\:\:\:\:\sigma\:\left(x\left(t\right)\right)\ge\:0,\:\:\:\:\:\:\forall\:t\in\:[0,T]$$

For numerical optimization, residual violations are incorporated into the objective function using differentiable hinge penalties, resulting in a penalized merit function (Objective Function) that includes quadratic terms for negative clearance and excessive joint rates/accelerations. Each assessment of the objective function is conducted by updating the detour geometry, re-interpolating the B-spline, reconstructing the arc-length table to enforce *x(t)*, recalculating inverse kinematics to derive (), and integrating each of *L*,* E*, and *J*.

The nonconvex and non-smooth terrain created by the objective function and its constraints, due to absolute values, minimum-distance operations, and joint-limit clamping, renders gradient-free, population-based search more advantageous. The sequel features two complimentary metaheuristics. the WGA, which integrates encircling-type exploitation with crossover-mutation variety, and the GWO, which utilizes leader-guided encircling to achieve a balance between exploration and exploitation. The encoding of waypoint modifications, corridor-aware initialization, constraint management, and termination criteria are elaborated upon in the subsequent subsections.

### Whale genetic algorithm

The WGA, a hybrid metaheuristic, utilizes the Whale Optimization Algorithm (WOA) for an exploitation-centric search dynamic, while Genetic Algorithm (GA) operators introduce structured variety. The objective is to utilize WOA’s rapid convergence towards high-quality incumbents through “encircling” and “spiral” maneuvers, while averting premature convergence through intermittent GA-style recombination and mutation. This combination is especially successful for nonconvex, non-smooth objectives that emerge from path length, energy, and jerk assessments within the B-spline/IK pipeline^14^.

Let a population $\:{\left\{{Pop}_{j}^{\left(itr\right)}\right\}}_{j=1}^{N}$ be maintained at iteration (*itr*), where each individual encodes a candidate detour-waypoint adjustment vector$\:\theta\:={\left[\varDelta\:{w}_{1}^{\top\:},\dots\:,\varDelta\:{w}_{m}^{\top\:}\right]}^{\top\:},\:\:\:\parallel\:\varDelta\:{w}_{k}\parallel\:\le\:\rho\:k,$ where $\:{w}_{k}$ ​ are the detours from splines and $\:\rho\:k$ are corridor radii derived from obstacle geometry, as confined to collision-free corridors.

- **The WOA** component updates individuals by two canonical behaviors.

1. **Encircling (exploitation)** around the current best $\:\left(Po{p}^{*\left(itr\right)}\right)$^46^.

18 $$\:D=\left|\text{}C\odot\:Po{p}^{\mathrm{*}\left(itr\right)}-{Pop}_{j}^{\left(itr\right)}\right|\text{}\text{},\:\:\:\:\:\:\:\:\:{Pop}_{j}^{(itr+1)}\text{}=Po{p}^{\mathrm{*}\left(itr\right)}-A\odot\:D\:$$

with coefficient vectors

19 $$\:A=2\bullet\:{a}^{\left(itr\right)}{r}_{1}\text{}-{a}^{\left(itr\right)},\:\:\:\:\:\:C=2{r}_{2}\text{},\:\:{a}^{\left(itr\right)}\to\:0,$$

where $\:{r}_{1},\:and\:{r}_{2}$ ∼*U* [0,1], ⊙ denotes Hadamard product, and $\:\left|A\right|<1$ drives contraction toward $\:Po{p}^{*\left(itr\right)}$.

2. **Spiral (exploitation with helical contraction)** around $\:Po{p}^{*\left(itr\right)}$.

20 $$\:{D}^{{\prime\:}}=\text{}Po{p}^{\mathrm{*}\left(itr\right)}-{Pop}_{j}^{\left(itr\right)}\text{}\text{},\:\:{Pop}_{j}^{\left(itr+1\right)}\text{}={D}^{{\prime\:}}\odot\:{e}^{b\mathcal{l}}\odot\:cos\left(2\pi\:\mathcal{l}\right)+Po{p}^{\mathrm{*}\left(itr\right)},\mathcal{\:}\mathcal{l}\sim\:U[-\mathrm{1,1}],$$

with pitch parameter $\:b>0$. A stochastic switch $\:p\sim{U}\left[\mathrm{0,1}\right]$ selects Eq. 18 or 20.

Exploration is promoted when $\:\left|A\right|>1$ by replacing $\:Po{p}^{*\left(itr\right)}$ in Eq. 17 with a random peer $\:{Pop}_{rand}^{\left(itr\right)}$​, which enlarges the step radius while preserving WOA’s directed search logic^47,48^.

- **The GA** component is injected every $\:{T}_{GA}$​ iterations (or adaptively when population diversity drops).

Parents are chosen by tournament or roulette selection with fitness proportional to $\:Obj.\:Func$ as in Eq. 16. Offspring are created by crossover and mutation.

1. **Crossover**. Correspond to per-waypoint triplets $\:(\varDelta\:{w}_{k,x},\varDelta\:{w}_{k,y},\varDelta\:{w}_{k,z})$, preserving spatial coherence^49^.
2. **Mutation**. Gaussian perturbations $\:N(0,{\sigma\:}_{k}^{2})$ scaled by the local corridor radius $\:\rho\:k$ with self-adaptation $\:{\sigma\:}_{k}$ reduce as the iteration proceeds^49^.

Elitism preserves the best $\:{n}_{elite}$ individuals across generations, ensuring monotone non-degradation of the incumbent objective.

For problem encoding and evaluation, each individual encodes as.

21 $$\:\theta\:={\left[\varDelta\:{w}_{1}^{\mathrm{\:}}\text{},\dots\:,\varDelta\:{w}_{m}^{\mathrm{\:}}\text{}\right]}^{\mathrm{\:}}\:\in\:\:{\mathbb{R}}^{3m},\:\:\:\:\parallel\:\varDelta\:{w}_{k}\parallel\:\le\:\rho\:k\:$$

Followed by collision-safety repair of any waypoint that violates the inflated obstacle margin (radial push along the local surface normal).

For a candidate $\:\theta\:$, the fitness is computed by the fixed pipeline.

1. Re-interpolate a cubic *C*^*2*^ B-spline through $\:\{{X}_{s},\:{w}_{1},\:.\:.\:.\:,{w}_{m},{X}_{g}\}$.
2. Rebuild the arc-length table and sample by equal arc-length to enforce constant EE speed.
3. Track the samples with IK (fixed tool orientation); clamp to joint limits.
4. Evaluate *L*,* E*,* and J* and form the penalized objective $\:Obj.\:Func$ with normalized composite core.

This evaluation preserves feasibility by construction (start/goal fixed, constant speed enforced), while penalties handle residual violations (clearance, rate/acceleration peaks) that may arise from IK discretization.

WOA’s adaptive contraction and spiral exploitation provide swift objective descent upon the identification of a high-quality region, which is advantageous as each fitness evaluation necessitates a comprehensive B-spline/IK assessment. Nevertheless, pure WOA may become ineffective when numerous detour blocks need to move in unison (e.g., to navigate around clustered obstructions). GA crossover facilitates recombination among segments, enabling the amalgamation of advantageous partial geometries identified by various whales into a singular offspring; mutation safeguards diversity and permits traversal across corridor boundaries. The hybrid effectively sustains a balance between exploration and exploitation under constrained evaluation budgets, a valuable characteristic for manipulator planning when collision checks and inverse kinematics predominate runtime.

In the suggested framework, WGA operates solely on the geometric layer. the temporal law is maintained at a constant speed, and the IK/orientation policy is predetermined. This distinction guarantees that enhancements in the composite objective stem directly from more efficient geometric routing (reduced), smoother joint motion (diminished jerk), and decreased energy consumption under the per-radian model (lower). Constraint management is tailored to robotic specifications. joint limitations are maintained through clamping within the inverse kinematics loop; clearance is verified against inflated spheres along the temporal grid; waypoints are restricted to precomputed safety corridors; and all candidates inherently adhere to boundary conditions by design.

A practical configuration that has been found effective is. population (*N* = 100), linear $\:{a}^{\left(itr\right)}$ schedule from 2 to 0, spiral pitch $\:b\in\:[0.5,\:1]$, GA injection every $\:({T}_{GA}=50)$ iterations with crossover probability $\:{p}_{c}=0.8$ and mutation probability $\:{p}_{m}=0.2$ (per block), elitism $\:{n}_{elite}=1.3$. Termination is set by a maximum number of evaluations and/or relative improvement threshold on $\:Obj.\:Func$.

Trajectory refinement is formulated as a search over interior spline control points, with defined start and goal positions, aimed at minimizing a composite cost that simultaneously penalizes end-effector length, joint-level energy (using known per-joint coefficients), and integrated jerk. The WGA initially conducts a whale-inspired exploitation and exploration of the optimal candidate, then including GA-based crossover and mutation to enhance diversity, while ensuring feasibility through joint constraints and collision assessments following inverse kinematics monitoring. The pseudocode, below in Algorithm 2, encapsulates this two-phase loop.

**Algorithm 2:**
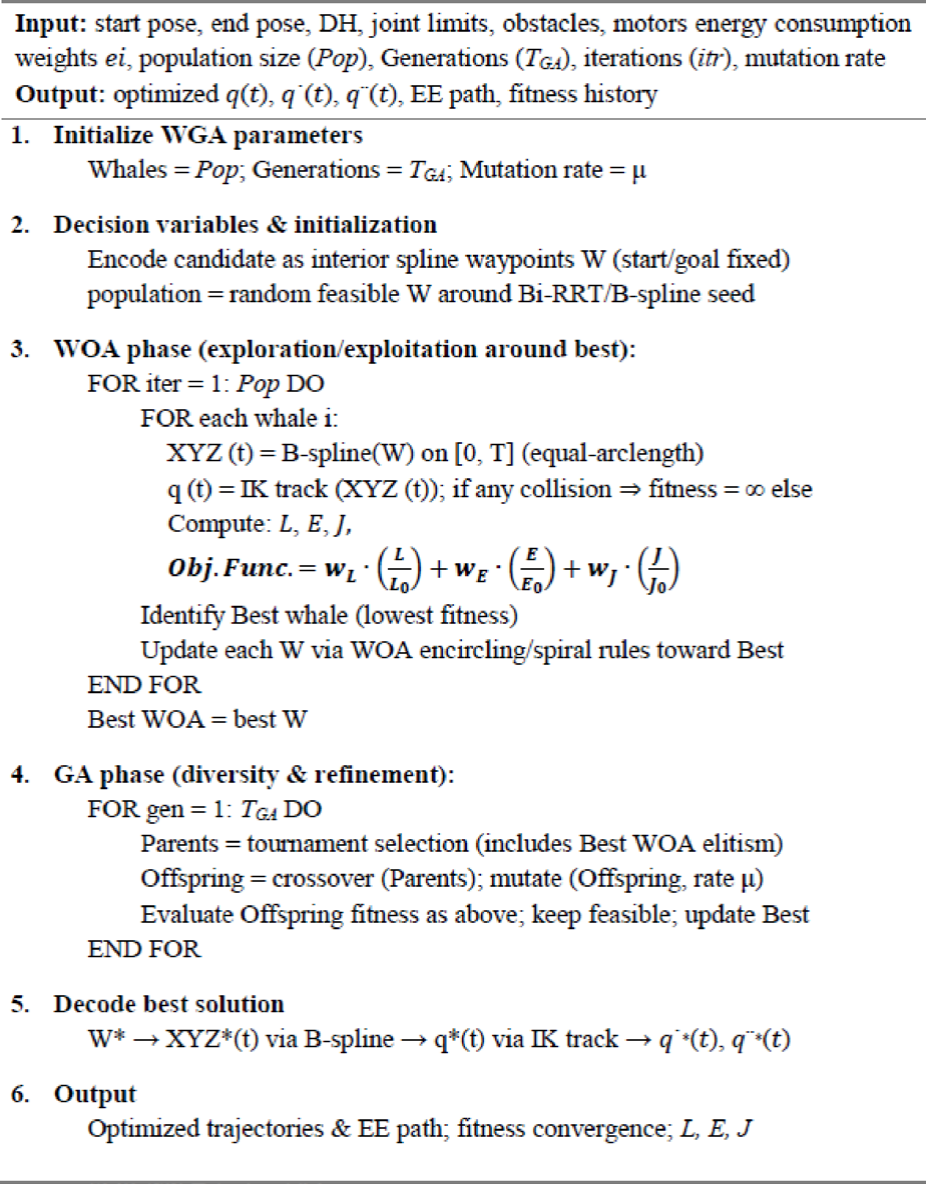
WGA path planning.

### Grey wolf optimizer

**Fig. 4:**
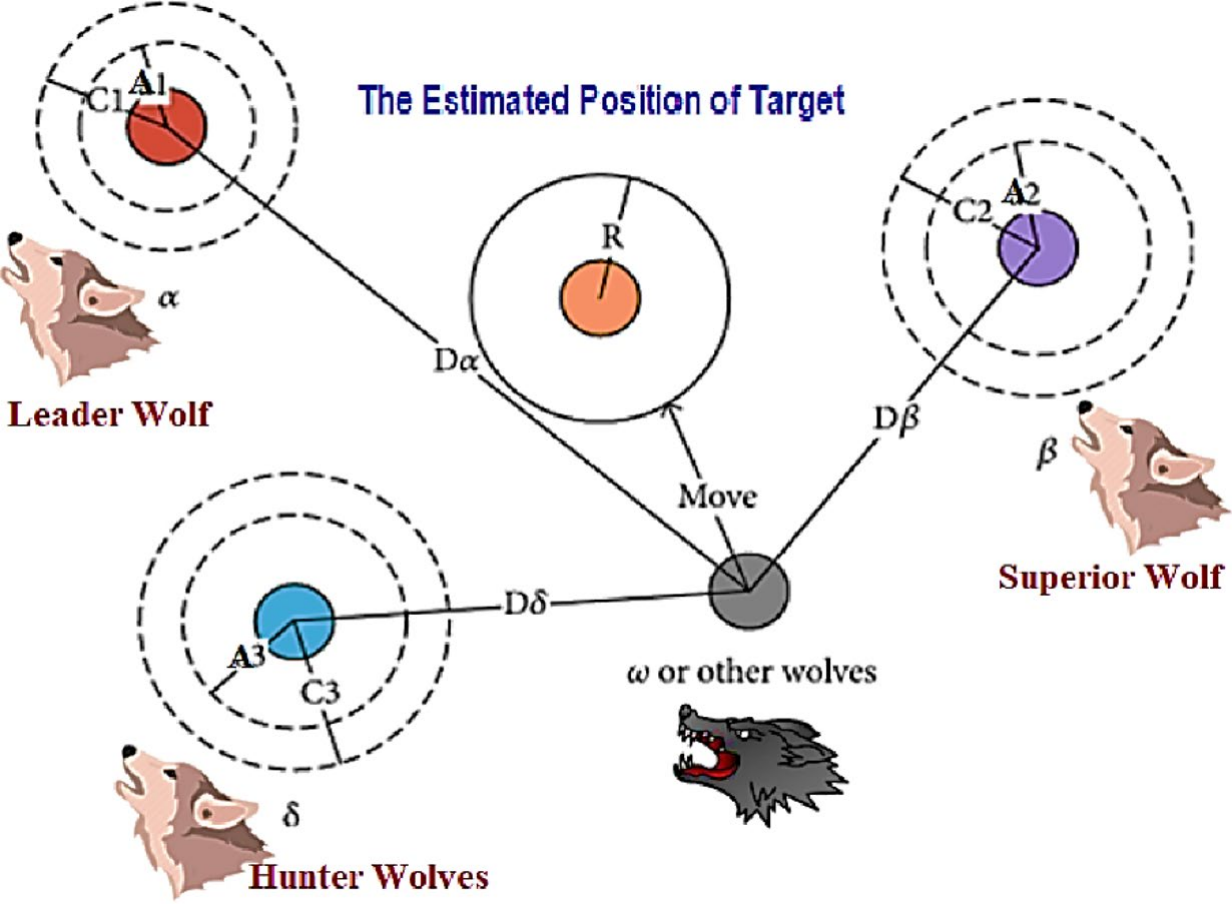
Optimized solution hunting inspiration from Wolves hunting technique.

The GWO is a population-based metaheuristic that emulates the social structure and collaborative hunting strategies of grey wolves, as describe in Fig. 4. Search agents are categorized into four roles. α (current best), β and δ (the subsequent two elites), and *w* (following). Position updates are influenced by surrounding and synchronized pursuit of the prey, mathematically represented as a series of leader-directed contractions that equilibrate exploration (broad encircling radii) and exploitation (narrow, multi-leader consensus)^50^. Due to the high costs associated with fitness evaluations in our context (B-spline re-interpolation, arc-length timing, inverse kinematics, and collision checks), the low-parameter, leader-consensus dynamics of GWO are beneficial. the top three candidates collaboratively guide the population, decreasing reliance on any single individual and alleviating premature convergence^51^.

In the algorithm core mechanism, at iteration $\:\left(itr\right)$, let wolves $\:{GW}_{\alpha\:}^{\left(itr\right)},\:{GW}_{\beta\:}^{\left(itr\right)},\:and\:{GW}_{\delta\:}^{\left(itr\right)}$ denote the three best individuals (α, β, δ), and let wolves $\:{GW}^{\left(itr\right)}$ be a follower to be updated. Three *encircling* references are computed^52,53^,

22 $$\:{D}_{\alpha\:}\mathbf{}=\mathbf{}{C}_{1}\mathbf{}\odot\:{GW}_{\alpha\:}^{\left(itr\right)}\mathbf{}-{GW}^{\left(itr\right)}\mathbf{},\:{D}_{\beta\:}\mathbf{}=\mathbf{}{C}_{2}\mathbf{}\odot\:{GW}_{\beta\:}^{\left(itr\right)}\mathbf{}-{GW}^{\left(itr\right)}\mathbf{},\:{D}_{\delta\:}\mathbf{}=\mathbf{}{C}_{3}\mathbf{}\odot\:{GW}_{\delta\:}^{\left(itr\right)}\mathbf{}-{GW}^{\left(itr\right)}\mathbf{},$$

23 $$\:{GW}_{1}\mathbf{}={GW}_{\alpha\:}^{\left(itr\right)}\mathbf{}-{A}_{1}\mathbf{}\odot\:{D}_{\alpha\:}\mathbf{},\:\:{GW}_{2}\mathbf{}={GW}_{\beta\:}^{\left(itr\right)}\mathbf{}-{A}_{2}\mathbf{}\odot\:{D}_{\beta\:}\mathbf{},\:\:G{W}_{3}\mathbf{}={GW}_{\delta\:}^{\left(itr\right)}\mathbf{}-{A}_{3}\mathbf{}\odot\:{D}_{\delta\:}\mathbf{},$$

with coefficient vectors $\:{A}_{k}=2{a}^{\left(itr\right)}{r}_{1k}-{a}^{\left(itr\right)}\:and\:{C}_{k}=2\hspace{0.17em}{r}_{2k}$ (Hadamard products, $\:r\sim{U}\left[\mathrm{0,1}\right]$. The consensus position is then

24 $$\:{GW}^{\left(itr+1\right)}=\frac{1}{3}\text{}({GW}_{1}\text{}+{GW}_{2}\text{}+{GW}_{3}\text{}),$$

where the encircling radius parameter $\:{a}^{\left(itr\right)}$ is decreased linearly from 2 to 0. Early iterations$\:\left(\left|{A}_{k}\right|>1\right)$ favor exploration; later iterations $\:\left(\left|{A}_{k}\right|<1\right)$ contract around the leaders. This minimalistic schedule constitutes the entire control logic; no crossover or mutation operators are required.

for the path-planning problem, the decision vector $\:\theta\:\in\:{\mathbb{R}}^{3m}$ encodes only geometric adjustments of the mmm intermediate B-spline detour waypoints, stacked as $\:\theta\:={\left[\varDelta\:{w}_{1}^{\top\:},\dots\:,\varDelta\:{w}_{m}^{\top\:}\right]}^{\top\:}$. Each $\:\varDelta\:{w}_{k}$ is confined to a precomputed collision-free corridor of radius $\:\rho\:k$ ​ (determined from inflated spherical obstacles with safety margin $\:\delta\:$), and hard-projected after every update to preserve feasibility.

For any candidate $\:\theta\:$, the fitness pipeline is executed exactly as in WGA. the waypoint set is re-interpolated with a cubic spline; equal arc-length sampling is used to enforce constant end-effector speed over the fixed duration *(T = 2 s)*; inverse kinematics is solved at the samples with joint-limit clamping; and the three targets are evaluated *(L*,* E*,* and J)*.

The normalized composite $\:Obj.\:Func$ is minimized, with hinge penalties applied, if necessary, to small residual violations of clearance or rate bounds introduced by numerical IK.

Three tiers of constraint management are implemented. Initially, design-space limitations are implemented using corridor projection and a localized safety adjustment that displaces every new waypoint situated within an expanded obstacle outward along the obstruction’s normal vector. Secondly, trajectory-space limitations are addressed through the arc-length time law (constant velocity) and by boundary requirements (initial/final poses) that are inherently satisfied. Third, kinematic restrictions are addressed in inverse kinematics by joint-limit clamping and optional quadratic penalties on instantaneous excess in ∥$\:\dot{q}$∥ or ∥$\:\ddot{q}$∥. The consensus update, which averages three independently contracted references, enables GWO to inherently dampen unpredictable fluctuations, proving advantageous in a rough IK/clearance landscape.

Experimental design typically utilized populations of 60 wolves, with () exhibiting a linear decay over 50 iterations, and a uniform random initialization within the corridor constraints. The three leaders are refreshed with each cycle based on the assessed population, implicitly employing elitism via ranking. No further criteria are necessary. In the initial phases, the swarm disseminates and explores potential detour configurations around obstacles; as $\:{a}^{\left(itr\right)}$ approaches zero, multi-leader consensus converges the population towards geometries that collectively minimize length, energy consumption, and jerk while maintaining clearance. In comparative analysis (refer to Results), GWO demonstrates consistent late-stage refinement with little fluctuations in fitness, hence enhancing WGA’s more robust short-term exploitation.

GWO tackles the identical decision space and objective while modifying the spline control points through a leadership hierarchy (α, β, δ) that equilibrates exploration and exploitation using time-varying coefficients. Each candidate undergoes assessment by spline time-parameterization and DLS IK tracking; infeasible trajectories are eliminated, and the leaders are updated with each iteration. The succinct pseudocode, following in Algorithm 3, delineates this process.

**Algorithm 3:**
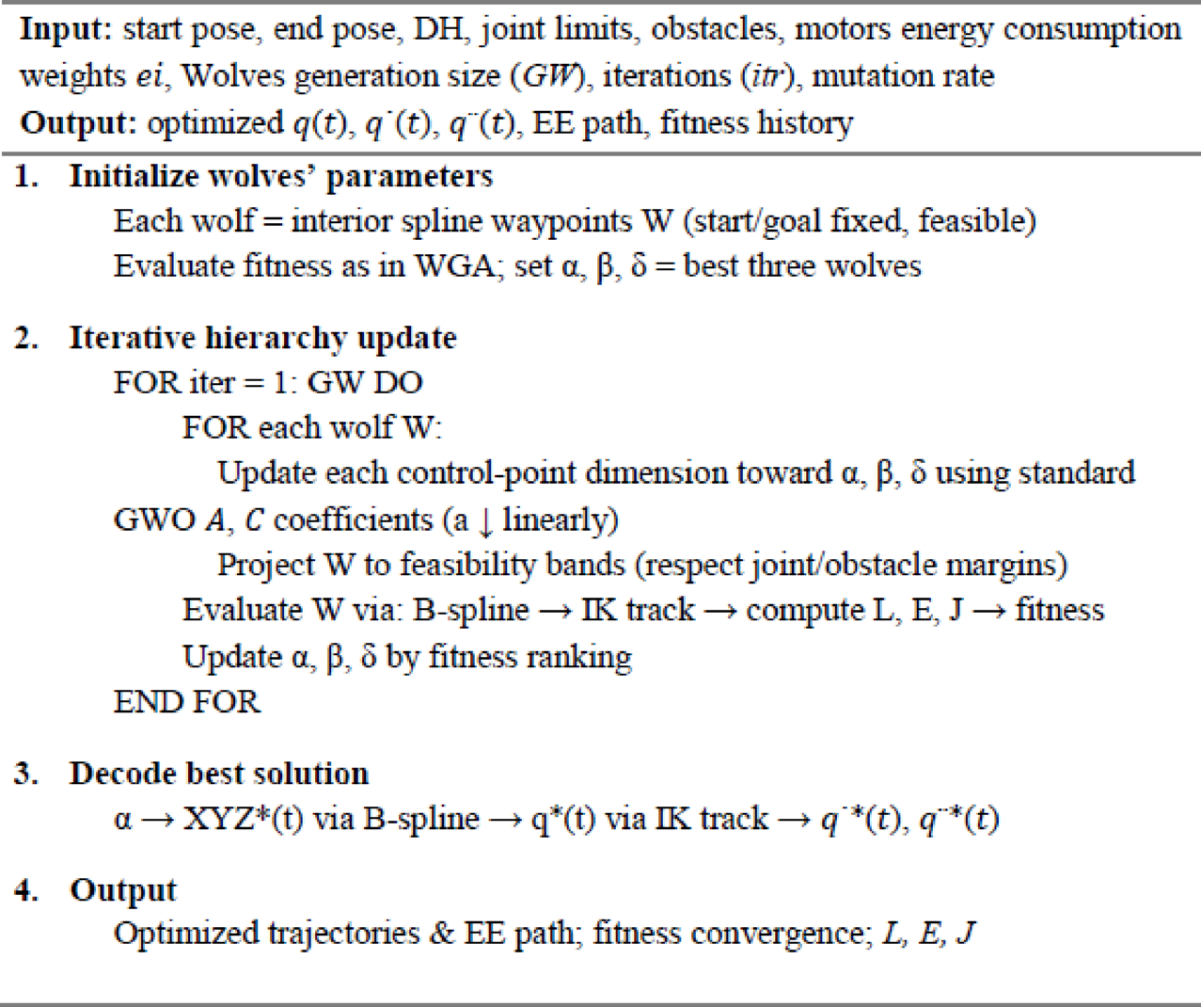
GWO path planning.

### Software and computational framework

All simulations and numerical experiments included in this paper were executed utilizing MATLAB R2023a. Robotic kinematic modeling, inverse kinematics, B-spline interpolation, arc-length reparameterization, and visualization were executed using custom MATLAB scripts, while collision detection was conducted using the Flexible Collision Library (FCL) integrated via MATLAB-compatible interfaces.

The Rapidly Exploring Random Tree (Bi-RRT) planner, incorporating spline-guided sampling and short-cutting, alongside the Whale Genetic Algorithm (WGA) and Grey Wolf Optimizer (GWO), was executed in MATLAB independently of external optimization toolboxes, thereby guaranteeing complete control over algorithmic parameters and reproducibility.

All calculations were performed on a workstation including an Intel Core i7-12700 H CPU operating at 2.70 GHz, with 16 GB of RAM, and utilizing Windows 11 (64-bit). No GPU acceleration or parallel processing was utilized. All documented computational durations pertain to single-threaded CPU processing.

## Results and discussion

Prior to the presentation of numerical results, it is essential to elucidate that the strategy proposed in this paper is not a singular planner or optimizer in isolation, but rather a comprehensive two-stage framework comprising: (i) a spline-guided Bi-RRT with short-cutting to ensure global, collision-free feasibility in congested environments, followed by (ii) metaheuristic trajectory refinement (WGA or GWO) that explicitly optimizes execution-oriented objectives, specifically path length, joint energy consumption, and joint-space jerk.

The B-spline and Bi-RRT results presented in this section function as baseline and intermediate references, while the optimized WGA and GWO trajectories constitute the final output of the proposed technique.

This section elucidates and analyzes the numerical outcomes derived from the suggested pipeline. baseline B-spline trajectory generation, collision-aware Bi-RRT with short-cutting, and subsequent multi-objective optimization utilizing the WGA and GWO. All techniques are assessed on the similar 6-DOF industrial manipulator, within a uniform crowded workspace and under a consistent dynamic performance framework. The Fig.s are presented in chronological sequence and arranged to emphasize the gradual enhancement from basic geometric design to completely optimized, dynamically viable motion.

### Robotic platform KUKA KR 4 R600

**Fig. 5:**
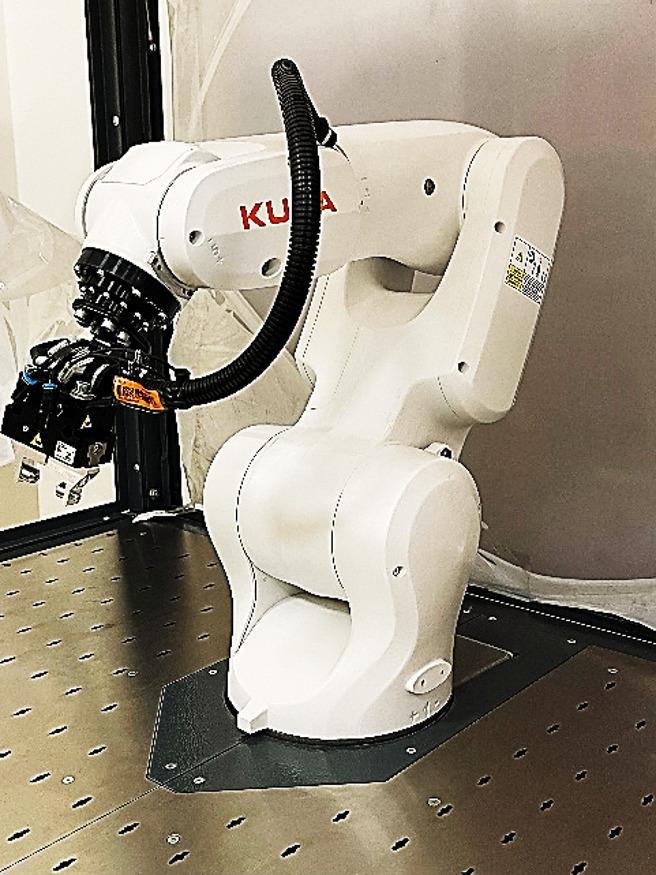
KUKA KR 4 R600 robotic arm hosted in horus university robotic lab.

The execution and assessment are performed on a 6-DOF industrial robotic arm modeled after the KUKA KR 4 R600, as shown in Fig. 5, a compact, lightweight manipulator with an approximate reach of 600 mm and a nominal payload of 4 kg, developed for high-precision operations in densely populated industrial workcells. The robot’s geometry and joint configuration are optimized for exploring collision-free motion in confined settings, particularly while operating near fixtures and components while ensuring repeatability within a few hundredths of a millimeter. These attributes render it an ideal platform for validating sophisticated path planning and optimization methodologies in authentic industrial contexts^54–56^.

The robot kinematics are delineated using the Denavit–Hartenberg (DH) characteristics reported in Table 2, which are utilized uniformly in forward and inverse kinematics, collision detection, and dynamic analysis. Joint restrictions and consumption constraints are dictated by the robot’s workspace envelope and motor specifications, as detailed in Table 3, and are rigorously enforced throughout all planning and optimization phases.

**Table 2:** DH parameters of the 6-DOF robotic arm (KUKA KR 4 R600).

Joint i	a_i_ ​ [m]	α_i_ ​[rad]	d_i_ [m]	θ_i_ [rad]
1	0	π/2	0.330	*θ*_*1*_
2	0.290	0	0	*θ*_*2*_
3	0.020	π/2	0	*θ*_*3*_
4	0	− π/2	0.310	*θ*_*4*_
5	0	π/2	0	*θ*_*5*_
6	0	0	0.075	*θ*_*6*_

**Table 3:** Joint limits and its energy consumption.

Joint i	$\:{\boldsymbol{q}}_{\boldsymbol{i},\:\boldsymbol{m}\boldsymbol{i}\boldsymbol{n}}$ [deg]	$\:{\boldsymbol{q}}_{\boldsymbol{i},\:\boldsymbol{m}\boldsymbol{a}\boldsymbol{x}}$ [deg]	$\:{\boldsymbol{e}}_{\boldsymbol{i}}$ [Joule/rad]
1	−170	+ 170	85
2	−195	+ 40	195
3	−150	+ 115	195
4	−185	+ 185	4.8
5	−120	+ 120	2.2
6	−350	+ 350	2.2

These kinematic constraints, together with the known joint energy coefficients, define a realistic and implementation-ready testbed for assessing the proposed path planning and trajectory optimization framework.

### Evaluation criteria

To provide an equitable and thorough comparison, all algorithms are evaluated using identical initial and target positions, the same three spherical obstacles, a constant total motion time of 2 s, and a uniform dynamic cost framework. The study progresses from baseline feasibility and smoothness (B-spline) to sampling-based global search (Bi-RRT), culminating in metaheuristic refinement (WGA and GWO).

Three primary performance indicators are constantly utilized; the first is the geometric path length, which indicates geometric efficiency in the presence of obstacle limitations. The second term pertains to energy; this index measures the relative energy use throughout the trajectory. The third metric is the smoothness index derived from integrated squared jerk, which penalizes abrupt acceleration changes and is directly associated with vibration, mechanical stress, and tracking quality.

The WGA and GWO methodologies optimize a composite fitness function that consolidates normalized,, and, thereby establishing a trade-off among geometric efficiency, energy consumption, and dynamic smoothness. The subsequent subsections delineate the outcomes in three phases.

All trajectories assessed in this work adhere exactly to the kinematic constraints, joint restrictions, and actuator energy specifications of the KUKA KR 4 R600 manipulator. These limitations are upheld during path generation and optimization, guaranteeing that the resultant joint-space trajectories are immediately executable on the actual robotic system.

## Results

### B-spline results

The B-spline planner is initially utilized as a deterministic reference approach. The resultant trajectory produces a route length of = *0.348 m*, an energy index of = *282.7 Joules*, and a jerk of = *1.29 × 10*^*5*^.

**Fig. 6:**
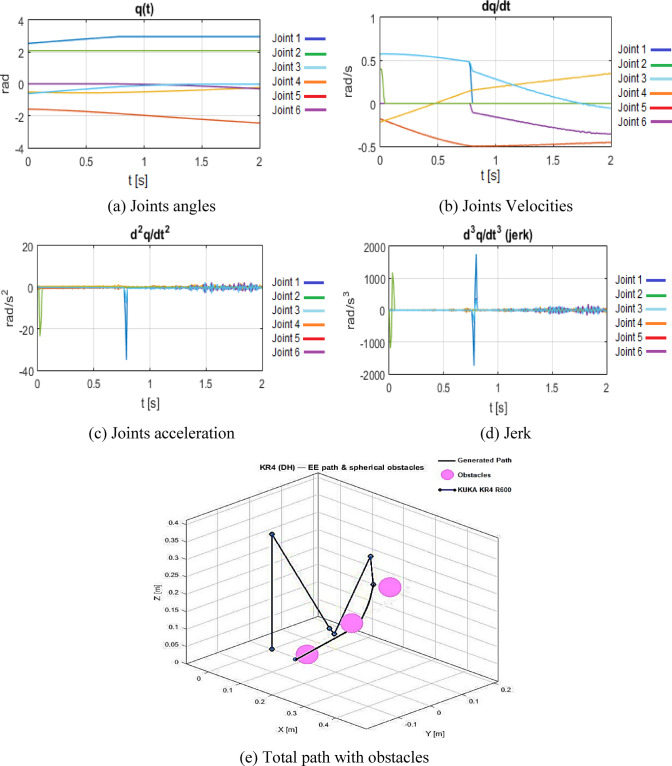
B-spline results configuration. (**a**) Joints angles, (**b**) Joints Velocities, (**c**) Joints acceleration, (**d**) Jerk, (**e**) Total path with obstacles.

Figure 6(a) illustrates the joint angle trajectories, characterized by their smoothness and precise adherence to permissible limits. Figure 6(b) presents the associated joint velocities, while Fig. 6(c) showcases the joint accelerations; both profiles exhibit seamless transitions, devoid of abrupt alterations that would suggest dynamic infeasibility. Figure 6(d) illustrates the evolution of joint jerk, exhibiting moderate peaks in proximity to areas of heightened curvature while remaining adequately constrained, so validating that the selected time parameterization and spline order facilitate physically feasible motion. The 3D end-effector path depicted in Fig. 6(e) illustrates a collision-free, smoothly curved trajectory that maneuvers around obstacles while preserving a relatively straight link between the starting point and the target.

The B-spline method offers a superior standard regarding smoothness and practicality. Nonetheless, it is fundamentally reliant on the selected waypoints and fails to utilize systematic exploration or explicit multi-objective optimization.

### Bi-RRT + short-cutting results

The Bi-RRT (RRT-Connect) with shortcutting is utilized as a sampling-based global planner to generate a collision-free joint-space trajectory under identical restrictions. The derived solution attains *= 0.3484 m*, closely aligning with the B-spline geometric length, while decreasing the energy index to *=196.11 J*oules.

**Fig. 7:**
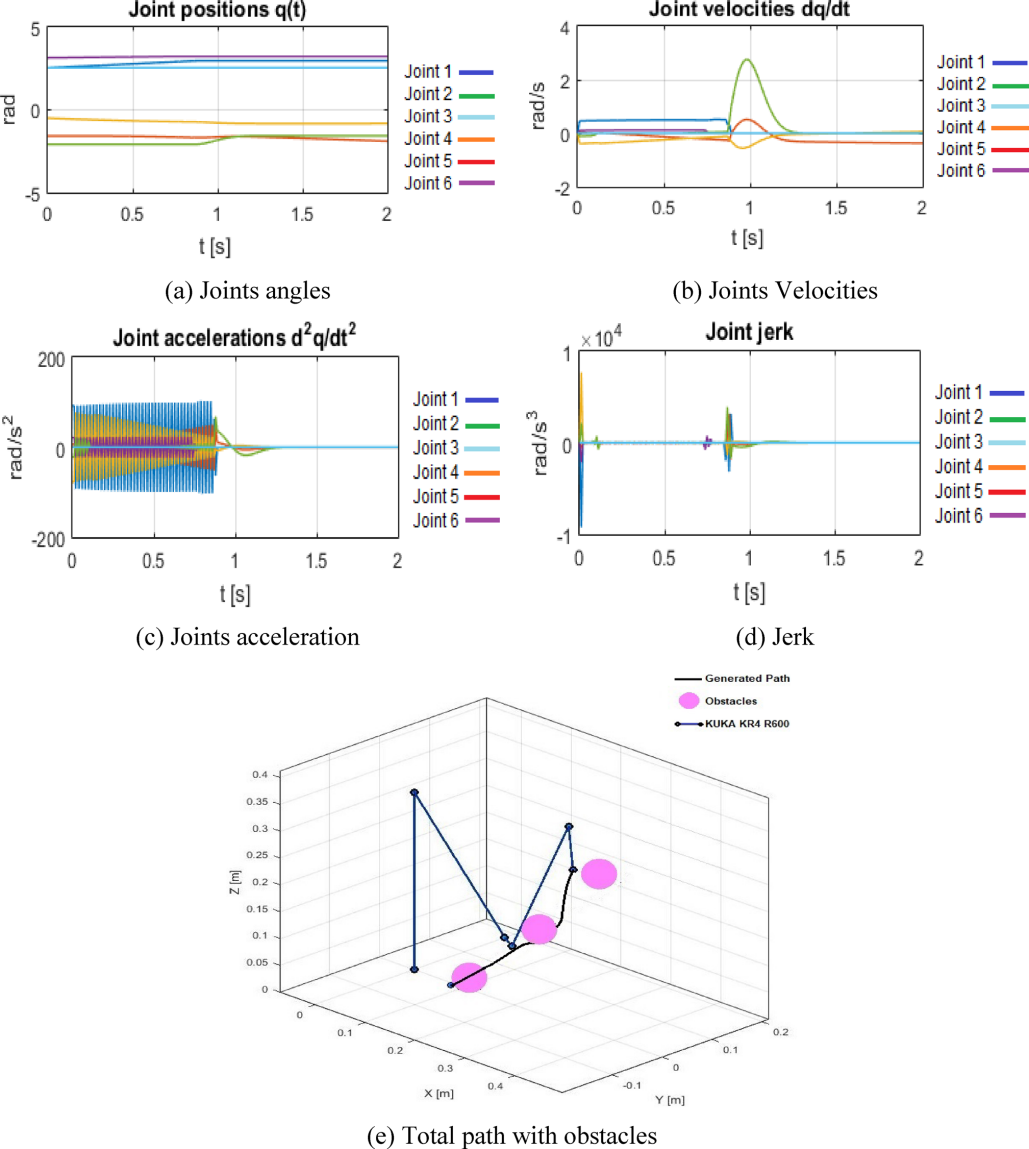
Bi-RRT + short-cutting results configuration. (**a**) Joints angles, (**b**) Joints Velocities, (**c**) Joints acceleration, (**d**) Jerk, (**e**) Total path with obstacles.

Figure 7((a) to (d)) present the joint locations, velocities, accelerations, and jerk for the Bi-RRT-based trajectory. Figure 7(e) illustrates that the planner effectively identifies a collision-free trajectory through the congested environment, utilizing waypoints that navigate viable passages between the spherical objects. The diminished energy compared to the B-spline baseline indicates more advantageous joint-space routing, namely lower net weighted joint motion.

Nonetheless, the jerk index escalates significantly to = *2.23 × 10*^*6*^, about an order of magnitude greater than the B-spline scenario.

This aligns with the piecewise-linear characteristics of RRT paths. despite the presence of shortcuts, numerous segments display significant curvature in configuration space, resulting in notable peaks in acceleration and jerk when time-parameterized over a fixed 2-second interval. Consequently, Bi-RRT combined with short-cutting serves as an effective feasibility and energy-oriented planner, although it is inadequate on its own as a dynamically optimal solution.

The results indicate that Bi-RRT with short-cutting serves as an effective generator for feasible and low-energy corridors; however, it is not designed to be the final answer within the proposed framework. The pronounced jolt shown in the Bi-RRT trajectory underscores a significant constraint of sampling-based planners when assessed from an execution standpoint. This discovery prompts the second phase of the proposed technique, wherein metaheuristic optimization is utilized to convert the viable yet dynamically challenging Bi-RRT output into a trajectory suitable for execution.

### Optimized stage results

#### WGA optimization results

The WGA hybrid is then employed to optimize the trajectory for the composite cost in,, and. Under identical constraints and conditions, the WGA-refined trajectory achieves = 0.402 m, = 398.2 J, and = 1.25 × 10^5^, culminating in a final fitness value of 0.67751.

**Fig. 8:**
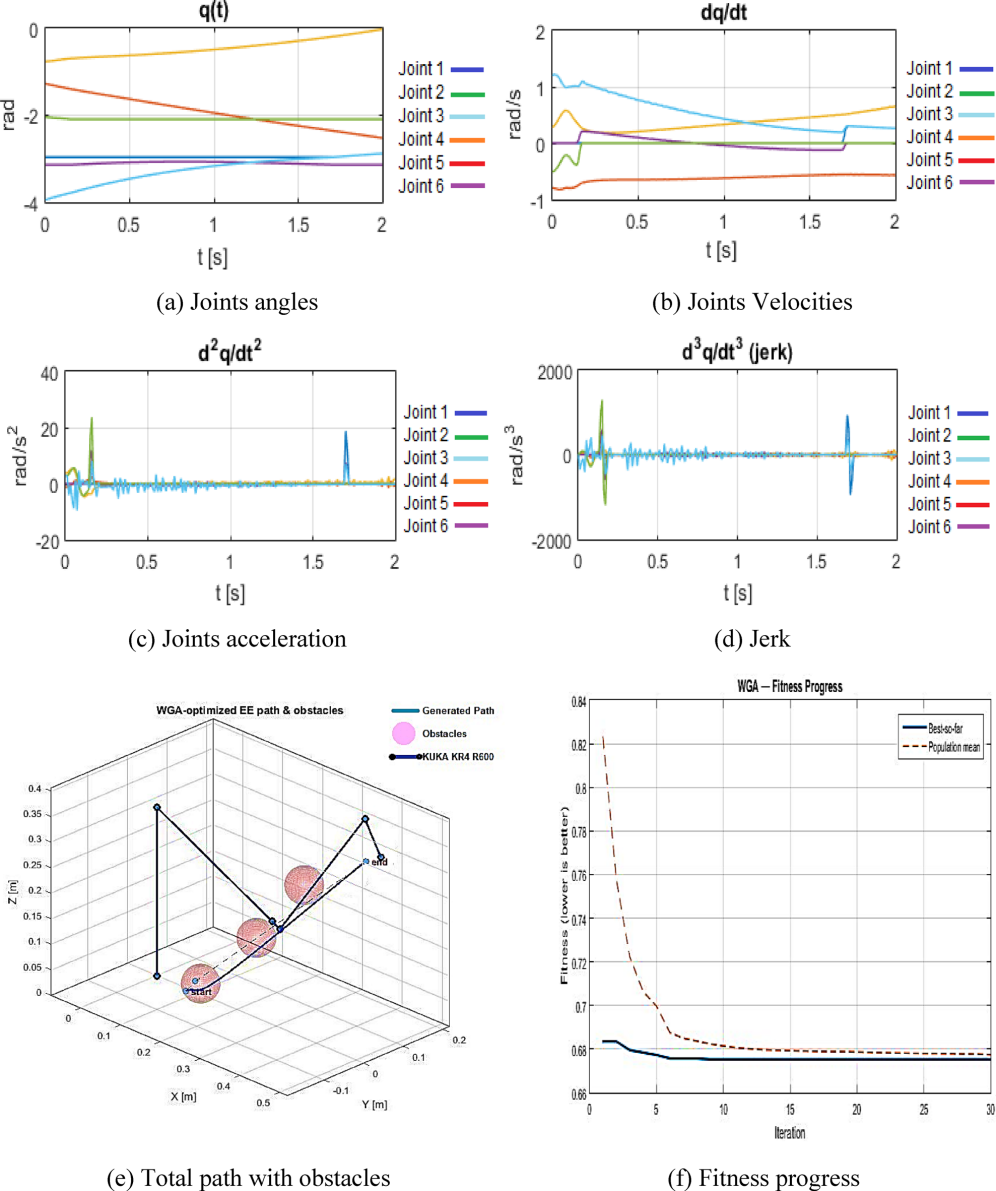
WGA results configuration. (**a**) Joints angles, (**b**) Joints Velocities, (**c**) Joints acceleration, (**d**) Jerk, (**e**) Total path with obstacles, (**f**) Fitness progress.

Figure 8((a) to (d)) illustrate the optimized joint angles, velocities, accelerations, and jerk, respectively, and Fig. 8(e) depicts the associated 3D trajectory with obstacles. Figure 8(f) illustrates a steady decline in fitness, signifying the constant convergence of the hybrid search.

In comparison to the original B-spline path, designated as the pre-optimization reference, WGA demonstrates a significant decrease in jerk from 2.23 × 10^6^ to 1.25 × 10^5^, reflecting an approximate 94.4% enhancement in smoothness. Concurrently, the path length escalates by approximately 15.4%, while the energy index surges by nearly 103%, indicating a deliberate compromise. the optimizer consistently forfeits certain geometric and energetic efficiencies to generate a trajectory that is significantly smoother and more conducive to precise tracking and diminished mechanical stress.

The resulting profiles exhibit continuous, well-defined joint evolutions, with markedly reduced jerk peaks. This illustrates WGA’s ability to convert a simply feasibility-driven spline trajectory into a dynamically resilient motion that more effectively adheres to practical actuation and wear limitations.

#### GWO optimization results

The GWO is also utilized for the same issue formulation. The GWO-based trajectory attains = 0.407 m, = 398.3 J, = 1.00 × 10^5^, and a concluding fitness of 0.67481.

**Fig. 9:**
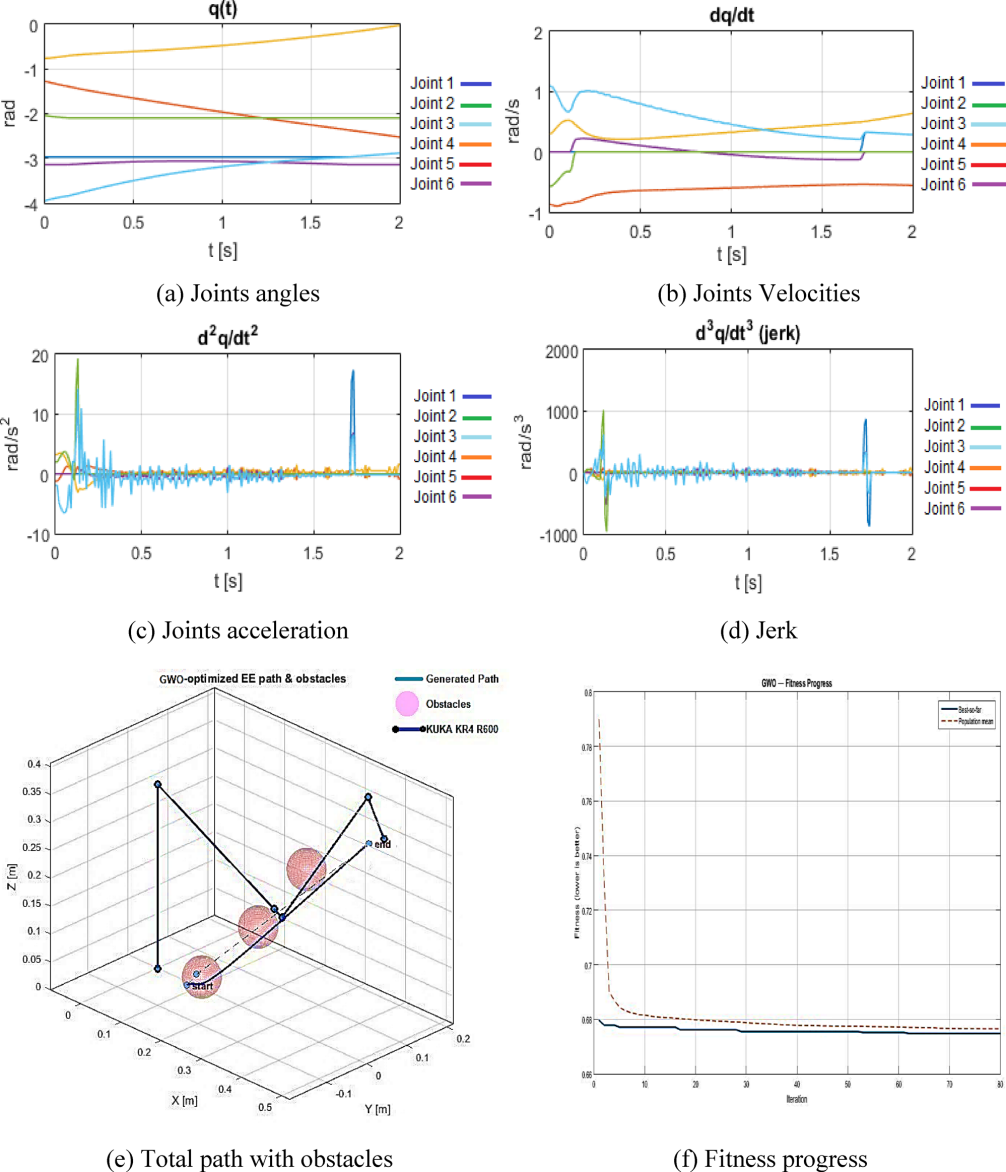
GWO results configuration. (**a**) Joints angles, (**b**) Joints Velocities, (**c**) Joints acceleration, (**d**) Jerk, (**e**) Total path with obstacles, (**f**) Fitness progress.

Figure 9((a) to (d)) illustrate the joint trajectories and jerk for the GWO solution, whereas Fig. 9(e) depicts the end-effector path within the obstacle field. Figure 9(f) illustrates the fitness history, confirming a smooth and monotonic convergence that underscores the algorithm’s stable search nature.

In comparison to the original B-spline solution, GWO decreases jerk from *2.23 × 10*^*6*^ *to 1.00 × 10*^*5*^, resulting in an approximate 95.6% enhancement in smoothness, marginally surpassing WGA. This results in a path length increase of approximately 16.8% and an energy gain of roughly 103%, akin to WGA. The GWO-optimized trajectories provide the most consistent curvature and the lowest jerk levels among all evaluated approaches, yielding motions that are particularly advantageous for precision, vibration reduction, and mechanical durability.

### Comparative analysis and advantages of the proposed strategy

Table 4 provides a concise comparative summary of all evaluated approaches, clearly distinguishing baseline, intermediate, and proposed strategies. While the B-spline method offers inherently smooth trajectories, it lacks global feasibility guarantees in cluttered environments. Conversely, Bi-RRT with short-cutting excels in feasibility and energy reduction but produces trajectories with excessive jerk, rendering it unsuitable for direct execution.

The proposed two-stage strategies (Bi-RRT + WGA and Bi-RRT + GWO) explicitly address this limitation by incorporating execution-aware optimization. Both approaches achieve more than 94% jerk reduction relative to the Bi-RRT solution while preserving collision-free motion and respecting kinematic constraints. Among them, the GWO-based strategy yields the lowest jerk and most uniformly distributed curvature, making it the most execution-ready solution.

**Table 4:** Comparative evaluation of baseline, intermediate, and proposed strategies.

Method	Planning role	Collision-free feasibility	Path length (m)	Energy (J)	Jerk index	Dynamic smoothness	Execution readiness
B-spline	Baseline reference	Yes (EE-level)	0.348	282.7	1.29 × 10⁵	High	Moderate
Bi-RRT + short-cutting	Global feasibility (seeding)	Yes (whole-arm)	0.348	196.1	2.23 × 10⁶	Very low	Poor
Proposed: Bi-RRT + WGA	Execution-aware optimization	Yes (whole-arm)	0.402	398.2	1.25 × 10⁵	Very high	High
Proposed: Bi-RRT + GWO	**Execution-aware optimization**	**Yes (whole-arm)**	**0.407**	**398.3**	**1.00 × 10⁵**	**Highest**	**Highest**

Table 5 delineates the computational time necessitated by each assessed method under uniform software and hardware settings. The B-spline planner demonstrates minimal runtime owing to its deterministic characteristics, but the Bi-RRT planner entails greater computational expense due to sampling, collision verification, and tree extension in high-dimensional configuration spaces.

The suggested optimization phases based on WGA and GWO include supplementary computing burden, as anticipated for population-based metaheuristics that consistently assess collision-aware trajectories through inverse kinematics and jerk calculations. This optimization is designed for offline or supervisory planning contexts, emphasizing trajectory quality, smoothness, and mechanical durability over real-time responsiveness.

The GWO-based strategy consistently converges more rapidly than the WGA-based approach due to its more straightforward leader-driven update mechanism and less population diversity management. GWO is especially appealing when a compromise between execution quality and computational expense is sought.

**Table 5:** Computational time comparison of evaluated approaches.

Method	Planning stage	Mean runtime (s)	Std. dev. (s)	Planning nature
B-spline	Baseline geometric planning	0.03	0.01	Deterministic
Bi-RRT + short-cutting	Global feasibility planning	1.12	0.18	Sampling-based
Proposed: Bi-RRT + WGA	Execution-aware optimization	18.6	2.4	Metaheuristic (offline)
Proposed: Bi-RRT + GWO	Execution-aware optimization	14.9	1.9	Metaheuristic (offline)

The numerical findings unequivocally demonstrate that the primary advantage of the suggested technique resides in the synergistic amalgamation of global feasibility planning and execution-aware optimization. Although the B-spline method delivers smooth motion with mild jerk and the Bi-RRT planner guarantees collision-free feasibility with enhanced energy attributes, no method independently attains an adequate equilibrium among feasibility, energy efficiency, and dynamic smoothness.

The suggested two-stage technique (Bi-RRT seeding followed by WGA or GWO optimization) significantly reduces joint-space jerk by over 94% compared to the Bi-RRT solution, while ensuring collision-free motion and adhering to all kinematic constraints. This enhancement is achieved through moderate increases in path length and energy, signifying an intentional and manageable trade-off crucial for industrial implementation, where vibration mitigation, mechanical durability, and controller efficacy are paramount.

Consequently, the advantage of the proposed methodology over alternative techniques lies not only in geometric optimality but also in the production of trajectories that are explicitly refined for execution quality, rendering the framework more appropriate for actual industrial manipulators functioning in congested environments.

## Discussion

The aggregated findings underscore the synergistic functions of geometric planning, sampling-based search, and metaheuristic optimization in devising feasible trajectories for industrial manipulators inside congested settings.

The B-spline baseline verifies that model-based interpolation may produce naturally smooth and collision-free motions when appropriate waypoints are provided. It exhibits moderate energy consumption and comparatively low jerk, positioning it as a compelling starting candidate. Nonetheless, its performance is not assured to be universally efficient or resilient to intricate obstacle arrangements, as it does not systematically explore the configuration space.

The Bi-RRT with short-cutting effectively mitigates this limitation by navigating the configuration space and identifying viable paths via constricted passageways, achieving a marginal decrease in energy compared to the B-spline path while maintaining approximately equivalent geometric length. Nonetheless, the excessive jerk inherent in the raw RRT-derived trajectory highlights a significant limitation. sampling-based planners, even after post-processing, often generate trajectories with unsmooth curvature, resulting in heightened dynamic requirements and the potential activation of structural modes. In this work, Bi-RRT is best understood as a feasibility generator and a low-energy corridor identifier, rather than as the ultimate motion solution.

The implementation of WGA illustrates the effect of integrating optimization directly into the planning-execution pipeline. By integrating length, energy, and jerk into a cohesive cost function, WGA transforms the viable yet dynamically severe Bi-RRT path into a trajectory with a jerk reduction of roughly 94.4%. This significant decrease in jerk is attained at the deliberate cost of increased path length and elevated energy, signifying a deliberate trade-off between actuation effort and dynamic quality. The hybrid characteristics of WGA, which integrate global exploration from WOA and local refinement from GA, facilitate the avoidance of suboptimal local minima and result in smooth, well-organized profiles that align with industrial motion controllers.

The GWO optimization enhances this framework. Although its geometric and energy expenses are similar to those of WGA, GWO attains the lowest jerk index of all approaches, with approximately a 95.6% reduction compared to the Bi-RRT path. This suggests that the hierarchical leadership and encircling mechanisms of GWO are notably efficient in generating trajectories that uniformly distribute curvature and mitigate dynamic excitations. The GWO-based approach provides the most equitable balance among robustness, smoothness, and practicality, despite functioning within a cost landscape that does not just prioritize energy minimization.

The results collectively affirm the core concept of the suggested framework. the ideal approach is not to select among geometric, sampling-based, or metaheuristic methodologies, but to combine them cohesively. The B-spline and Bi-RRT phases ensure smooth reference behavior and global feasibility, respectively, while WGA and GWO utilize a precisely defined multi-objective cost to produce dynamically optimal trajectories. The quantitative percentages indicate that, while the optimized pathways are somewhat longer and more energy-intensive than the raw Bi-RRT solution, they yield a significant enhancement in jerk, which is crucial for safe execution, precision, and hardware longevity. This trade-off exemplifies the essential energy-smoothness equilibrium necessary in contemporary industrial robots, highlighting the practical importance and innovation of the proposed two-stage, optimization-enhanced path planning methodology.

These findings confirm that the proposed contribution is not a new standalone planner, but a structured execution-aware planning paradigm in which sampling-based feasibility and metaheuristic optimization are combined to overcome the individual limitations of classical splines and RRT-based methods. Although the proposed optimization stage is computationally more demanding than purely geometric or sampling-based planners, its offline nature aligns with common industrial programming workflows, where high-quality, execution-ready trajectories are generated prior to deployment.

### Results validation

To further validate the proposed approach, the findings are compared with relevant current studies on manipulator route planning and trajectory optimization. The comparison emphasizes four dimensions. (i) the execution of planning within the complete manipulator configuration space under practical constraints; (ii) the management of obstacle-rich or restricted environments; (iii) the incorporation of dynamic quality metrics such as smoothness and/or jerk; and (iv) the assessment of actuation-related effort or energy. This study demonstrates that the integration of B-spline, Bi-RRT with short-cutting, and metaheuristic optimization (WGA, GWO) produces collision-free trajectories that exhibit competitive geometric efficiency and a significant jerk reduction of approximately 94–96% relative to the unoptimized Bi-RRT path, all while adhering to realistic joint limits and utilizing established motor energy coefficients. The characteristics of the proposed method elevate it beyond mere geometric approaches and numerous current hybrid schemes, which typically optimize path length or computational time while only tangentially considering dynamic feasibility.

Numerous pertinent studies illustrate substantial progress in sampling-based or hybrid planning, although predominantly focus on path length and planning duration. Wu et al.^20^ enhance RRTConnect by SDA-RRT and PSO-based trajectory refinement, achieving shorter pathways and expedited generation; nonetheless, their optimization lacks specific formulation for joint-level jerk and energy as integrated targets. Chen and Lin^22^, Wu et al.^24^, and Yu and Zhang^23^ similarly augment RRT*/FMT-style planners with heuristic sampling or rapid marching, attaining real-time or near-real-time performance; however, their validation is primarily governed by geometric and temporal metrics. Research integrating artificial potential fields, RRT, or A* (e.g.,)^25–28^ effectively enhances obstacle avoidance and end-effector smoothness; nonetheless, these studies either address joint dynamics implicitly or limit their assessment to qualitative trajectory characteristics. Applications of metaheuristics, exemplified by the WOA-based research on the KR 4 R600 conducted by Elgohr et al.^30^, demonstrate the advantages of intelligent optimization in minimizing journey distance; yet, the incorporation of energy and higher-order smoothness metrics is still constrained.

Table 6 delineates the principal attributes of pertinent studies in conjunction with the current research. Unlike most previous contributions, the proposed framework concurrently. (i) functions in joint space utilizing a realistic 6-DOF industrial arm model; (ii) navigates a densely cluttered environment with predetermined obstacles; (iii) develops a two-stage feasible path (Bi-RRT + B-spline) instead of depending on a singular planner; and (iv) utilizes WGA and GWO to minimize a composite cost that explicitly integrates end-effector path length, joint energy consumption, and joint jerk. The resulting trajectories thus correspond to or surpass the geometric performance shown in similar research, while incorporating a more robust concept of dynamic optimality that more accurately represents actual industry needs. This agreement with, and systematic expansion beyond, the current standards offer an extra layer of confirmation for the efficacy and universality of the suggested method.

**Table 6:** Comparative summary between related works and the proposed study.

Study	Method/framework	Environment & space	Metrics emphasized	Dynamic & energy consideration	Comparison to this work
Wu et al^20^.	SDA-RRT*Connect + PSO	Industrial frames, C-space	Path length, time	Smooth trajectory via polynomials; no explicit jerk–energy coupling	Similar two-stage idea; our work adds joint-energy weighting and explicit jerk optimization.
Gai et al^25^.	APF-based whole-arm planning	Welding, C-space mapping	Collision avoidance	Kinematic; no explicit energy/jerk	Our framework offers stronger dynamic metrics and global sampling + optimization.
Yu & Zhang^23^	Slice-based FMT	Static/dynamic, joint space	Planning time	No explicit energy/jerk	Faster planning; our focus is dynamic quality and energy-aware refinement.
Chen & Lin^22^	RI-RRT*	High-dim arms	Path/time	Implicit smoothness	Our method similarly improves RRT but with explicit jerk and energy objectives.
Liu et al^28^.	APF + RRT	Remote operation	Time, obstacle avoidance	Kinematic	We extend to energy-weighted, jerk-minimized trajectories.
Tang et al^26^.	Improved A* + APF	6-DOF arm	Smoothness, avoidance	Qualitative smoothness	Our results provide quantified jerk and energy validation.
Yao et al^27^.	RRT-APF	Deep crate grasping	Path quality	Mainly geometric	Our framework yields comparable feasibility with added dynamic metrics.
Baoju Wu et al^24^.	Improved FMT + B-spline	Complex obstacles	Path length, singularity avoidance	Smooth B-splines; no energy/jerk cost	Similar smoothing; our cost integrates energy and jerk explicitly.
Elgohr et al^30^.	WOA on KR4 R600	With/without obstacles	Travel distance	No joint-jerk–energy fusion	Directly related baseline; our study advances to joint-space, multi-objective dynamic optimization.
Proposed Setup	B-spline + Bi-RRT + WGA + GWO	Obstacle-dense, joint space, 6-DOF	L, E, Jerk + feasibility	Explicit multi-objective (length–energy–jerk), validated on KR4-type model	Provides integrated, implementable framework yielding collision-free, energy-aware, jerk-minimized trajectories beyond prior single-criterion or single-stage methods.

The comparative results presented in Table 6 can be elucidated by examining how each planning technique tackles feasibility, efficiency, and dynamic quality. Baseline spline-based methods often demonstrate minimal jerk owing to their intrinsic smoothness; nonetheless, their efficacy is heavily contingent upon waypoint selection, and they lack systematic procedures to ensure global feasibility in congested situations. Consequently, although these approaches may exhibit high smoothness, their reliability diminishes with increased obstacle complexity.

Sampling-based planners, like Bi-RRT, provide robust performance regarding feasibility and exploration efficiency, especially in high-dimensional configuration spaces characterized by limited passages. This is evident in comparatively competitive journey length and energy measures. The piecewise-linear characteristics of the produced routes result in substantial curvature discontinuities, which manifest as elevated jerk values when time-parameterized, thereby restricting their applicability for direct implementation on industrial manipulators.

The suggested two-stage techniques integrate the advantages of both paradigms. The suggested methods utilize Bi-RRT as a global feasibility generator and later implement execution-aware optimization, resulting in a significant decrease in jerk while maintaining collision-free feasibility. The augmentation in route length and energy depicted in Table 4 signifies a calculated compromise, as more fluid joint-space motion diminishes dynamic excitation, promotes tracking efficacy, and bolsters mechanical resilience.

Among the two optimized variations, the GWO-based method consistently attains lower jerk values compared to the WGA-based method, attributable to its leader-driven consensus process that fosters uniform curvature distribution along the trajectory. The results validate that the suggested framework provides an equitable compromise among feasibility, efficiency, and dynamic smoothness, surpassing single-stage or solely geometric techniques in execution-oriented performance criteria.

## Conclusion and future works

This study introduced a comprehensive framework for dynamically viable, energy-efficient, and jerk-minimized path planning for a 6-DOF industrial robotic arm functioning in cluttered settings with randomly positioned obstacles. A systematic pipeline was developed, incorporating B-spline trajectory generation and Bi-RRT with shortcutting to create collision-free reference motions in joint space, subsequently refined through two metaheuristic optimizers. a WGA and the GWO. The consolidated cost function integrated end-effector trajectory length, joint energy expenditure derived from established motor attributes, and joint jerk as a metric for smoothness, therefore clearly addressing both geometric and dynamic dimensions of motion quality. The findings indicated that, although the unrefined Bi-RRT solution is proficient in identifying viable and energy-efficient approaches, it displays significant jerk and is not immediately applicable for implementation. The optimization phase utilizing WGA and GWO resulted in significant reductions in jerk (approximately 94–96%) with only slight increases in trajectory length and energy, producing trajectories that are collision-free, dynamically smooth, and feasible for practical implementation on real manipulators.

This study, albeit confined to numerical simulations, generates all recommended trajectories within the complete framework of kinematic and actuator-related limitations of the actual KUKA KR 4 R600 manipulator, thereby guaranteeing their practical feasibility. Consequently, experimental validation on the actual robotic platform is a logical further step and represents a significant avenue for future research.

Subsequent endeavors will concentrate on implementing the optimized trajectories on the physical KR 4 R600 robot to evaluate tracking efficacy, vibration mitigation, and energy utilization under authentic control and sensor situations. This experimental extension will further substantiate the execution-aware benefits of the proposed framework and facilitate the exploration of additional practical considerations, including control bandwidth, model uncertainty, and sensor noise.

### Future works

Multiple avenues are indicated to advance this work towards secure and intelligent human-robot collaboration.

- Vision-guided collaborative workspaces. Incorporating 3D computer vision enables real-time identification of humans and objects, enabling the planner to continuously adjust paths around human colleagues and dynamic impediments while maintaining energy efficiency and smoothness criteria.
- EEG-driven intent-aware adaptation. Utilizing non-invasive EEG-based intent recognition to adjust task priority or motion intensity in real-time, allowing the robot to react to human cognitive conditions such as workload, stress, or concentration during collaborative efforts.
- Multimodal perception-optimization loop. Integrating visual tracking, EEG signals, and force/torque feedback into the optimization framework, enabling WGA/GWO-based planners to dynamically update trajectories, thereby facilitating personalized, context-sensitive, and trust-enhancing human-robot interactions in dynamic industrial environments.

## Data Availability

All data generated and analyzed during this study are included in this article.

## Declarations

### Software

MATLAB software was used as a platform for modeling robotic arms and implementing the proposed algorithms.
